# Emulating the early phases of human tooth development *in vitro*

**DOI:** 10.1038/s41598-019-43468-0

**Published:** 2019-05-07

**Authors:** Jennifer Rosowski, Julia Bräunig, Anna-Klara Amler, Frank P. Strietzel, Roland Lauster, Mark Rosowski

**Affiliations:** 1 Technische Universität Berlin, Institute of Biotechnology, Department Medical Biotechnology, Gustav-Meyer-Allee 25, 13355 Berlin, Germany; 2 Institute of Experimental Pediatric Endocrinology, Charité - Universitätsmedizin Berlin, Corporate Member of Freie Universität Berlin, Humboldt-Universität zu Berlin, and Berlin Institute of Health, Berlin, Germany; 3 Cellbricks GmbH, Gustav-Meyer-Allee 25, 13355 Berlin, Germany; 4 Charité Center for Dental, Oral, and Maxillary Medicine, Aßmannshauser Str.4-6, 14197 Berlin, Germany

**Keywords:** Stem-cell biotechnology, Tissue engineering

## Abstract

Functional *in vitro* models emulating the physiological processes of human organ formation are invaluable for future research and the development of regenerative therapies. Here, a developmentally inspired approach is pursued to reproduce fundamental steps of human tooth organogenesis *in vitro* using human dental pulp cells. Similar to the *in vivo* situation of tooth initiating mesenchymal condensation, a 3D self-organizing culture was pursued resulting in an organoid of the size of a human tooth germ with odontogenic marker expression. Furthermore, the model is capable of epithelial invagination into the condensed mesenchyme, mimicking the reciprocal tissue interactions of human tooth development. Comprehensive transcriptome analysis revealed activation of well-studied as well as rather less investigated signaling pathways implicated in human tooth organogenesis, such as the Notch signaling. Early condensation *in vitro* revealed a shift to the TGFß signal transduction pathway and a decreased RhoA small GTPase activity, connected to the remodeling of the cytoskeleton and actin-mediated mechanotransduction. Therefore, this *in vitro* model of tooth development provides a valuable model to study basic human developmental mechanisms.

## Introduction

Regenerative therapies rely either on the development of instructing treatments that cure the injured tissue by the support of self-regenerative capacities *in situ* or the generation of transplantable native surrogate tissues or organs *in vitro*. Organoid technology provides a promising strategy to obtain tissues with the requisite quality. The technology is based on human stem or progenitor cells, that were cultivated in guiding conditions to induce cell differentiation and cell arrangement resulting in an *in vitro* surrogate with a high resemblance to the *in vivo* counterpart^1^. Additionally to regenerative medicine the human organoids are valuable tools to study developmental or pathological processes and for drug development or toxicity screening^2^. For regenerative purposes a combination of organoid generation *in vitro* and *in situ* regenerative induction is conceivable. The *in vitro* generated transplantable organoid resembles the destroyed organ at a specific developmental stage and terminally differentiates upon transplantation to the injured site, guided by the surrounding microenvironment of the healthy instructing tissue. The success of such combined therapies is entirely dependent on the emulation quality of the initiating process for organ development. Thus, the knowledge about the processes on the molecular level, cell-cell, and cell-matrix interaction as well as the spatio-temporal requirements has to be translated to the organoid development.

The process of mesenchymal condensation is a crucial initial step for the development of numerous organs in embryogenesis. Although it is moving more and more into the focus of attention of research on organogenesis, the exact mechanism how mesenchymal condensation is regulated remains unknown. Earlier research on this developmental step was focused on skeletal formation elucidating its key role in activation of tissue-specific genes^3^.

Gradients of signaling molecules, cell-cell and, cell-matrix interactions drive the gathering and synchronized differentiation of the mesenchymal cells during formation of many organs such as tooth, hair, feather, cartilage and bone, kidney, lung and many more^2–7^. Intracellularly, complex and multifactorial signaling cascades are induced due to the changed cell shape, cell size and transduced mechanical stimuli. Finally, these activated signaling cascades trigger the induction of a tissue specific set of genes followed by cellular differentiation and fate commitment.

During embryonic tooth organogenesis, the inductive ecto-mesenchymal condensate develops at the site of future tooth^7^. Reciprocal epithelial-mesenchymal interactions with the overlying dental epithelium are mediated by morphogenic gradients of, e.g. FGF, TGFß, Wnt^8,9^. As shown in rodent models and developmental studies, cellular differentiation along the odontogenic lineage by mesenchymal dental precursor cells include the gene expression of Msx1 and Msx2, Pax9 and Runx2^10–12^. Although there is much information on murine tooth development in literature, little information on the spatio-temporal markers of human differentiation is available. To meet these drawbacks, there is an urgent demand for a versatile and reliable human tooth organogenesis model *in vitro*.

Herein we report the scaffold-free simulation of mesenchymal condensation of adult human dental pulp cells (DPCs), resulting in a condensate with odontogenic marker expression and the capacity to induce epithelial invagination *in vitro*.

We isolate human dental pulp cells from extracted wisdom teeth as described earlier^13^, expand them and re-induce mesenchymal condensation in ultra-low attachment culture. The DPCs immediately begin to interact and actively apply a traction force to form a condensate. We believe that the process of formation of self-organized cell condensates represents an excellent model to study the first steps of human organogenesis, i.e. mesenchymal condensation and epithelial-mesenchymal interaction. Whole Transcriptome analysis confirms the supposition that the cells undergo the process of mesenchymal condensation *in vitro* and specific marker expression suggests the obtainment of odontogenic potential.

## Results

### Characterization of isolated cell from human dental pulp

**Figure 1:**
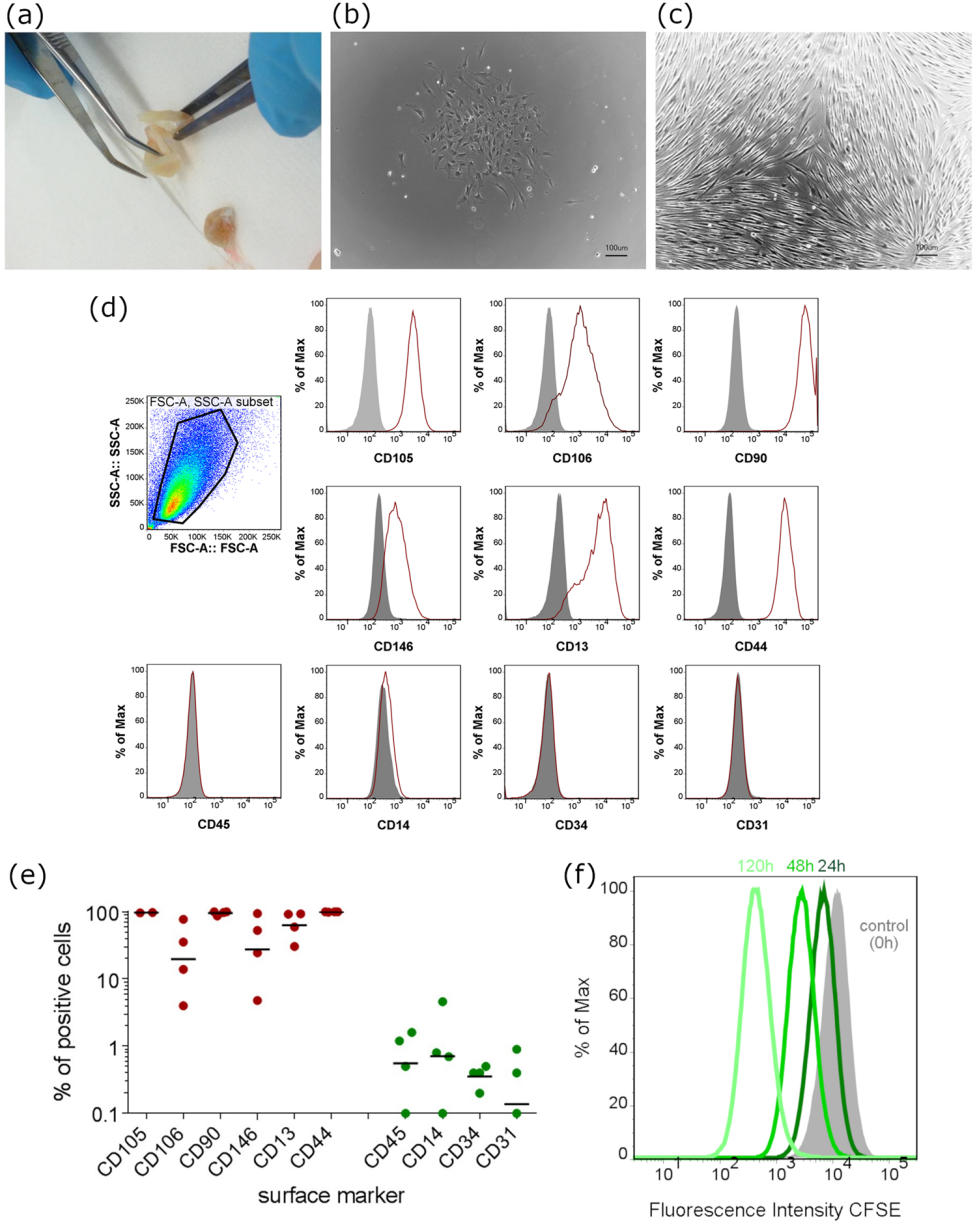
Isolation and characterization of dental pulp cells. (**a**) Isolation of dental pulp from tooth specimen, (**b**) colony formation of *in vitro* cultivated DPCs 72 hours after isolation, (**c**) confluent layer of DPCs in standard cell culture condition and clearly visible fibroblastic morphology of expanded cells. (**d**) Surface marker expression of expanded DPCs *in vitro* (N = 4). The histograms represent individual experiments where surface marker expression of individual donors was quantified by flow cytometry for the positive and negative mesenchymal stromal markers. Cells (P1) were positive for CD105, CD106, CD90, CD146, CD13 and CD44, but negative for CD45, CD14, CD34 and CD31. (**e**) The percentage of positive cells for the respective surface markers is depicted. The middle left graph shows the gating strategy for alive DPCs. (N = 4 biological replicates). (**f**) Population doubling was assessed by CFSE assay with measured time points of 0 hours, 24 hours 72 hours and 120 hours. CFSE intensity was lowered due to cell division and thereby a doubling time of 24.99 hours was calculated.

As previously reported human dental pulp cells (DPCs) are a population of ecto-mesenchymal origin with MSC characteristics *in vitro*. After isolation attached cells generated colonies of proliferating cells resulting in a confluent monolayer of spindle-shaped cells (Fig. 1a–c). The isolated cells used in this study exhibit a typical MSC surface marker expression profile in passage 1 (P1): CD90^+^, CD106^+^, CD44^+^, CD13^+^, CD146^+^ and CD45^−^, CD34^−^, CD14^−^, CD31^−^ as evaluated by flow cytometric analysis (Fig. 1d,e). In standard proliferative cell culture these cells exhibit a typical fibroblast-like morphology with a high proliferative capacity which was stable for more than 6 passages. Cell proliferation assay over 7 days was assessed by CFSE staining and indicates a doubling time of 24.99 ± 1.97 hours of human dental pulp cell in passage 4 (Fig. 1f).

### *In vitro* expansion of DPCs

**Figure 2:**
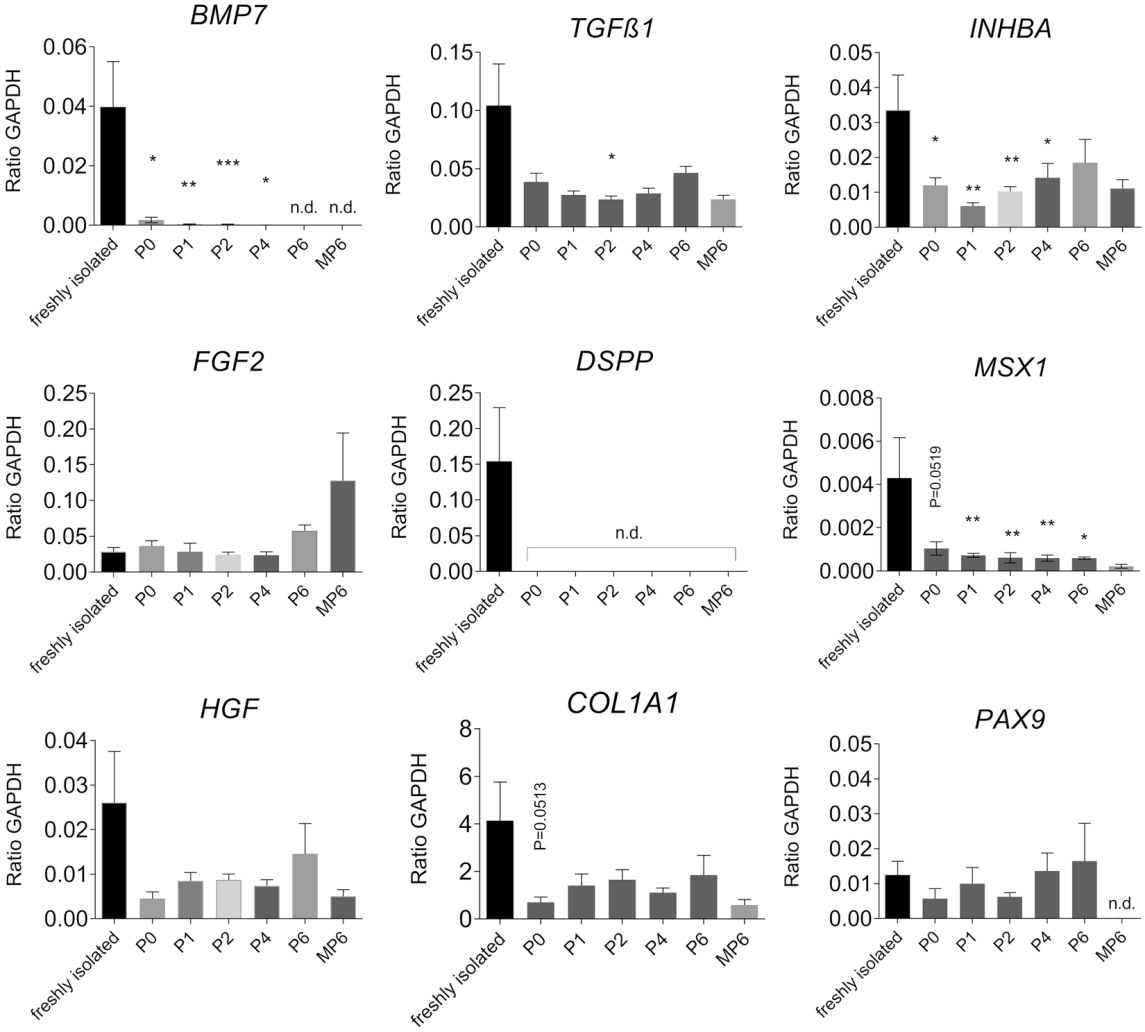
The monolayer expansion of DPCs is characterized by down regulation of marker molecules. RNA expression level as assessed by qRT-PCR of dental pulp cell marker genes (MSX1, PAX9, BMP7, TGFß1, INHBA, DSPP1, COL1A1, FGF2 and HGF) of freshly isolated DPCs from human wisdom teeth (freshly isolated) and of monolayer cultured DPCs of several passage (P0 to P6) and of mesenchymal stromal cells from human bone marrow in passage 6 (MP6). Expression values are relative to GAPDH expression (N ≥ 3 biological replicates; missing values were below detection limit – n.d.; ***P value < 0.001; **P value < 0,01; *P value < 0.05).

Marker gene expression of DPCs *in vitro* over 6 passages was assessed by RT-qPCR and compared to freshly isolated cells from dental pulp and passaged mesenchymal stromal cells (bmMSCs) isolated from bone marrow, respectively (Fig. 2). Except for the *FGF2* transcript, bone marrow MSCs of passage 6 (MP6) exhibit significantly lower or no detectable expression of all shown marker transcripts, *MSX1*, *PAX9*, *BMP7*, *TGFß1*, *INHBA*, *COL1A1*, *DSPP*, and *HGF* compared to freshly isolated DPCs. Upon *in vitro* cultivation, the majority of marker molecules were down modulated approximating the transcript levels of cultured bone marrow MSCs. For the transcription factor *MSX1*, a downregulation upon 2D adherent culture is visible compared to the expression level of freshly isolated cells. *PAX9* is not transcribed in bmMSC, but constantly high expressed in DPCs and not regulated upon monolayer culture over six passages. *BMP7* and *INHBA*, two members of the TGFß superfamily of signaling molecules, are significantly downregulated during cell culture. Also, the *TGFß1* ligand shows a downregulation in comparison to freshly isolated DPCs over monolayer culture time. Dentin Sialophosphoprotein (*DSPP*) and Collagen type I (*COL1A1*) represent two important downstream extracellular matrix molecule targets. The transcripts of DSPP are only slightly expressed or not detectable in monolayer expanded DPCs in monolayer culture whereas their expression directly after isolation from the dental pulp is high. *COL1A1* is slightly downregulated *in vitro* but expressed at a comparatively high level throughout the 2D culture and in bmMSCs. The growth factor FGF2 was constantly expressed independently of the *in vitro* amplification cultivation, while HGF exhibited lower RNA levels compared to freshly isolated DPCs.

### Differentiation capacity of DPCs *in vitro*

DPCs are described to be multipotent progenitor cells^14^. Therefore, here we show successful differentiation of our isolated DPCs to osteoblastic, adipogenic and neuronal precursor cells. Upon stimulation with osteoblast differentiation media a dramatic increase in mineralization as assessed via Alizarin Red staining was observed, and upregulation of Osteocalcin (OCN) and Osteopontin (OPN) transcripts could be detected by RT-qPCR (Fig. S1b). Upon adipogenic differentiation induction, an increase in the extent of lipid vesicles in the cytoplasm could be visualized by Oil Red O staining although the difference between the control population (cultured with standard DMEM with 10% FCS and Pen/Strep) was only weak. Nevertheless, gene expression of adipocyte-specific genes *FABP4* and *LPL* clearly shows a differentiation into the adipogenic lineage (Fig. S1a). DPCs cultured in neurogenic media significantly exhibit a changed morphology, expression of Nestin and Tuj-1 as confirmed by immunohistochemistry and upregulation of neuro-specific transcripts (TUJ1 and NES) (Fig. S1c).

### Mesenchymal condensation of DPCs *in vitro*

**Figure 3:**
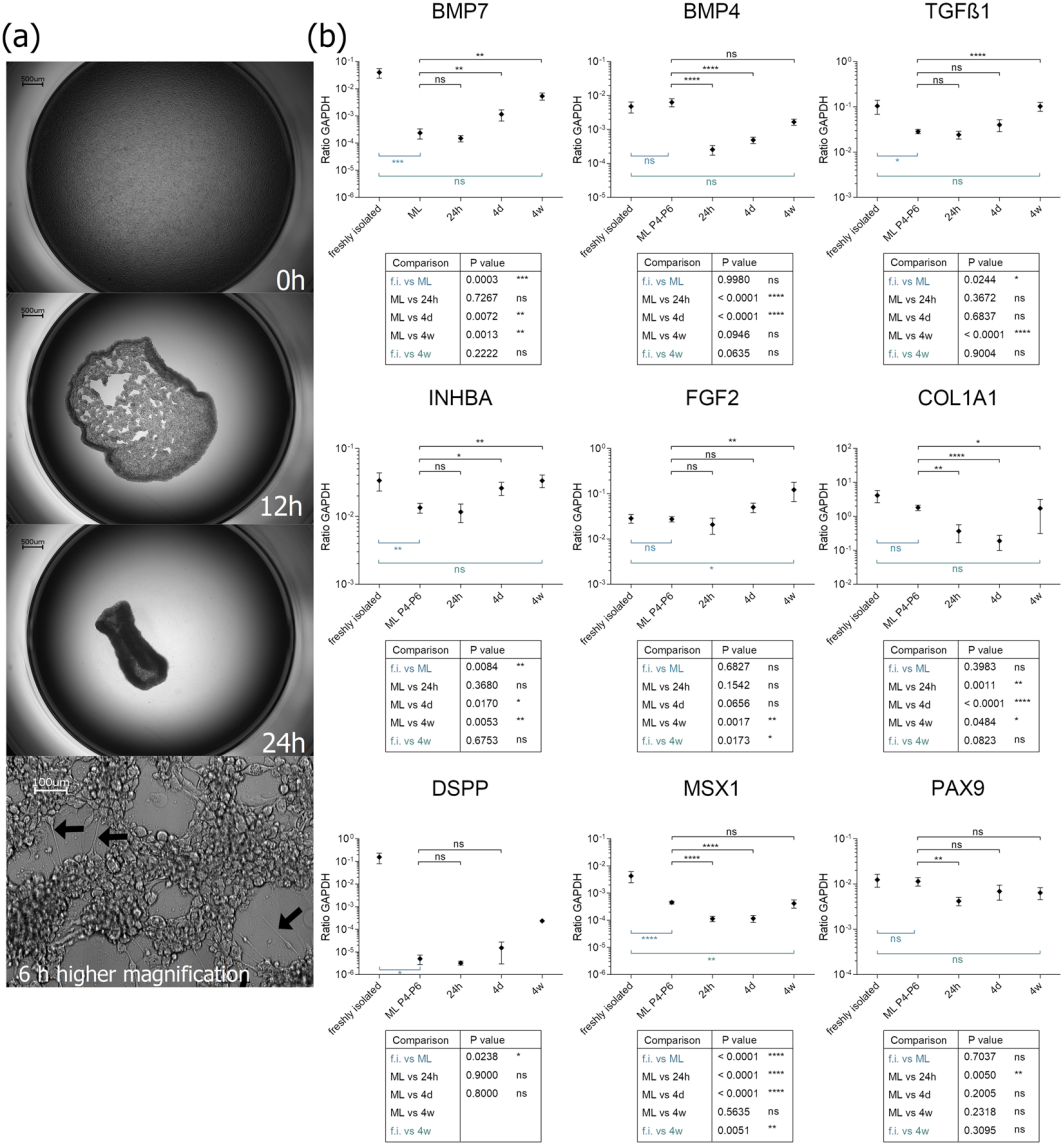
(**a**) Kinetic of ultra-low attachment culture of DPCs. After 6 hours, long cell protrudings are formed between the cells (lowest picture, black arrows). (**b**) Analysis of gene transcription level of chosen marker genes during the condensation process. Depicted are relative expression values of BMP7, FGF2, PAX9, INHBA, TGFß1 and COL1A1 of the DPC condensates at 24 hours (24 h), 4 days (4d) and 4 weeks (4w) after induction of condensation culture. For comparison also expression values of freshly isolated DPCs (f.i.) and monolayer DPCs in different passages (ML P2-P6) are presented. ns - not significant; *P < 0.05; **P < 0.01; ***P < 0.001; ****P < 0.0001. N ≥ 5 biological replicates.

To imitate the step of mesenchymal condensation *in vitro*, we cultured DPCs in a density of 10^6^ cells per ml in Ultra Low-Attachment culture vessels. Thereby, the cells are forced to interact with each other. After 4–6 hours the cells form long protrusions and interconnect with each other (Fig. 3a). The cells generate attracting and tension forces resulting in a dense aggregate after 24 hours of cellular condensation of 500 µm in diameter. (Fig. 3a and Supplemental Video S1).

Interestingly, the self-condensing 3D culture leads to an upregulated expression level of dental specific marker genes as assessed by RT-qPCR (Fig. 3b). The most prominent effects were observed in the expression kinetics of *BMP7*, Activin A (*INHBA*) and *TGFß1*. As described above *TGFß1* and *BMP7* are subjected to monolayer expansion with respect to downregulation of their transcripts. During 3D culture *TGFß1*, *BMP7* and Activin A are upregulated after 4 days of culture and reach an expression level comparable to directly isolated DPCs (statistically no significant difference calculated). Expression of Collagen type I (*COL1A1*) significantly decreases upon 3D culture over the time course of 4 days and slightly re-increased after 4 weeks of 3D culture. Also, the expression of *DSPP* elevated after 4 weeks in some culture replicates although this is not consistent for all donor cells. In several donors, the *DSPP* expression persisted below the detection level or was not existent. We analyzed expression levels of two dental-specific transcription factors *MSX1* and *PAX9*. The level of *PAX9* expression is constantly high in our cells, and only weak changes are detected after 24 hours of 3D cultures. The transcription “recovers” to the level of freshly isolated DPCs after 4 days. The expression of *MSX1* dramatically drops upon monolayer culture (described above) and even more in 3D culture. A very slight upregulation can be detected after 4 weeks of 3D culture but only to the initial monolayer level. The expression of *FGF2* is dramatically increased only in our 3D cultures and is 5 times higher after 4 weeks of 3D culture than in freshly isolated DPCs (Fig. 3b). The partially low expression ratios of the target molecules can be attributed to the exceptional high expression of the housekeeping gene GAPDH (compare RPM-values in Fig. S2).

The re-differentiation capacity of DPCs by the 3D condensation process prompted a comprehensive gene expression analysis. Initial odontogenic differentiation requires cytoskeletal rearrangement induced by mechanical compaction and traction forces^15^. Therefore, the very first signals, that are induced upon and in the course of the contraction during the *in vitro* condensation were of interest in the next experiment.

### Comprehensive gene expression analysis of the early condensation phase

To determine the most significant point in time, a short term kinetic of the low-attachment 3D culture was conducted. The time point with the most intriguing changes was defined after 6 h of condensation since the cell aggregation process was characterized by significant cellular morphological changes and differential expression of mesenchymal condensation marker molecules (data not shown). The transcriptome data were compared to the expression pattern of monolayer cultures. The analysis was performed with two donors for each condition (ML versus 6h_3D).

An average of 1.05 million 150-base long reads from each donor and condition were mapped to the human reference genome and assembled. The calculated RPM expression values were used for cluster analysis and to characterize the condensation process on the molecular and functional level.

**Figure 4:**
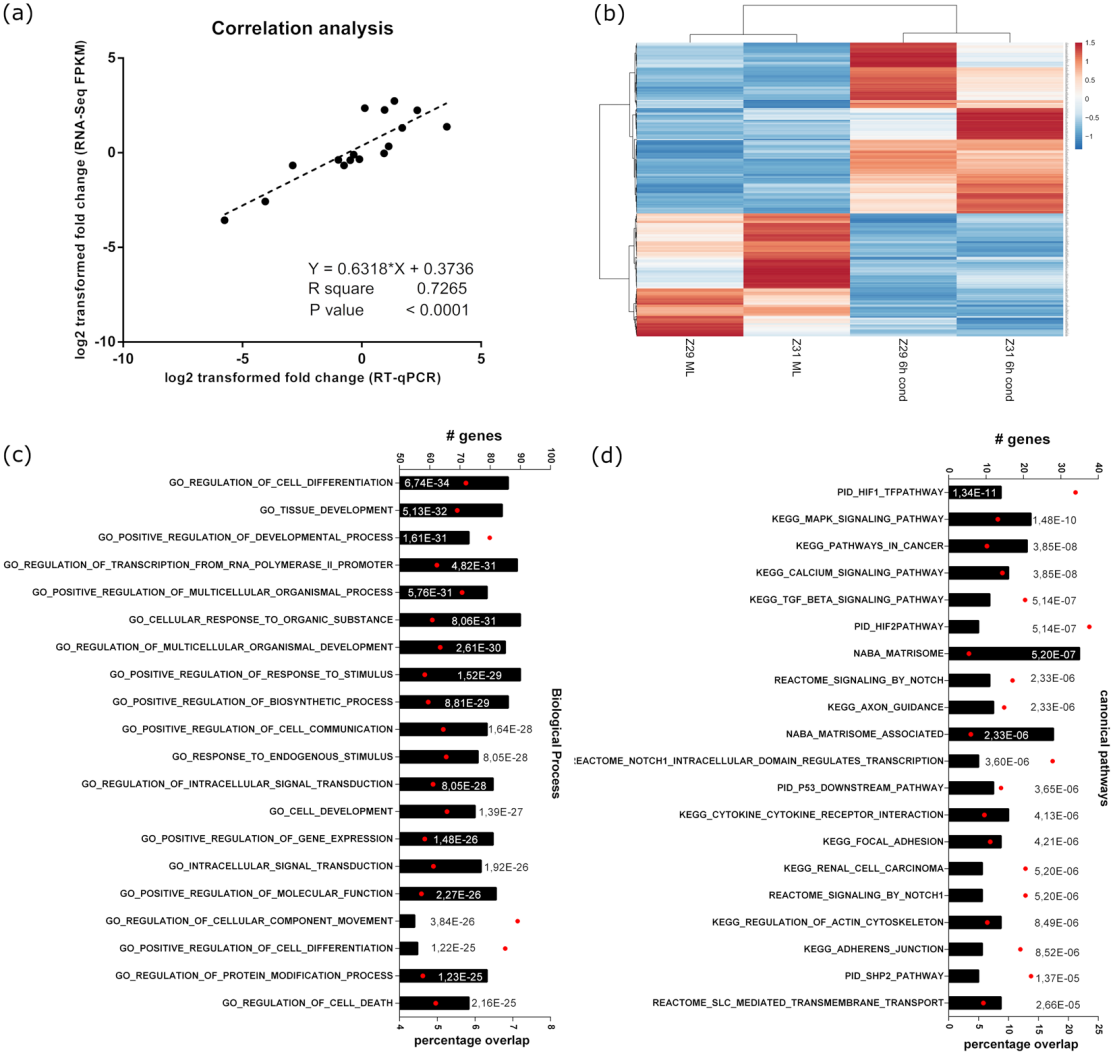
Comprehensive transcriptome analysis of the early DPC condensation. (**a**) Significant correlations between expressions of quantitative real-time PCR and RNA-Seq. Depicted are the log2 fold changes of seven genes, obtained by RNA-Seq (FPKM values) or by qRT-PCR (*Δ* Ct to GAPDH values). (**b**) Cluster analysis of differentially expressed genes between monolayer cultured (ML) or condensed (cond) DPCs according to DESeq2 algorithm (nominal P value < 0.05). 521 differentially expressed genes display even distribution of upregulated and downregulated genes. The color code represents the range between minimal (blue) and maximal (red) relative expression based on RPM values. (**c**,**d**) Differentially expressed genes were analyzed by Gene Set enrichment analysis. The top 20 over-represented gene ontology (GO) terms (FDR q-value < 0.05) for (**c**) ‘*biological process*’ as well as for the (**d**) ‘*canonical pathways*’ gene set is depicted. The diagrams plot the total number of genes overlapping in the differentially expressed genes and the ontology list (upper x-axis, # genes, black bars). Furthermore, the percentage of overlap (related to the group size to the GO term) is shown (lower x-axis, red dots) is depicted.

To assess the quality of the obtained data, we performed a correlation analysis by plotting the expression values obtained by qRT-PCR against the respective expression values obtained by NGS of selected marker genes. The results show that RNA-Seq data correlated well with the RT-qPCR data (Fig. 4a; P < 0.0001 and R^2^ = 0.727).

Interestingly, comprehensive cluster heatmap analysis of all expressed genes reveal the transcriptome properties of individual primary cells with donor intrinsic expression intensities characterized by donor-specific clustering (data not shown).

Differential Gene Expression Analysis by application of the DESeq2 algorithm resulted in 521 significantly differentially expressed genes (nominal P value < 0.05; |log2(fold change)| >1.5) with an almost even distribution of up and down regulated genes (Fig. 4b). To allocate affected biological processes, molecule classes and signal transduction pathways Gene Set Enrichment Analysis with a focus on particular issues like biological function or signal transduction pathway was performed. Depicted are the Top10 enriched GO terms in each ontology group assigned to molecular functions and canonical signal transducing pathways. Induction of DPC condensation produced mainly GO-terms connected to cell differentiation developmental processed and cell communication in the “biological process” set of genes (Fig. 4c).

The mesenchymal condensation associated pathways *TGFß signal transduction pathway (KEGG)* and *Regulation of actin cytoskeleton (KEGG)* emerged in the overrepresentation enrichment analysis in canonical pathway category (Fig. 4d). The three growth factors *TGFB1*, *TGFB2*, and *TGFB3* were significantly upregulated along with the TGFß specific signal transducer Smad3. Together with the downregulation of *BMP4* and the TGFß signaling specific inhibitor Smurf2 the data suggest a shift from BMP to TGFß signaling. Furthermore, the increased level of *INHBA* and the corresponding receptor molecule *ACVR1* emphasize the shift to Smad2/Smad3 mediated signal transduction. On the other hand, the BMP pathway specific inhibitors Noggin and Gremlin show decreased RNA levels, and the member of the BMP subfamily *BMP8A* was detected to be upregulated.

The Notch signaling pathway was deduced repeatedly by gene set enrichment analysis, especially in the REACTOME database context. A set of 13 genes related to Notch signaling are differentially expressed according to the DESeq2 analysis. Remarkably, both the ligand molecules *JAG1* as well as the receptor *NOTCH3* were defined to be higher expressed upon condensation induction and suggest an autocrine activation of the signaling. This assumption is emphasized by the downregulation of inhibitory co-repressors histone deacetylases (HDAC) 7 and 11 as well as the elevated levels of transcriptional targets like *HES1* and *HEYL* and the receptor processing molecule *FURIN*.

**Figure 5:**
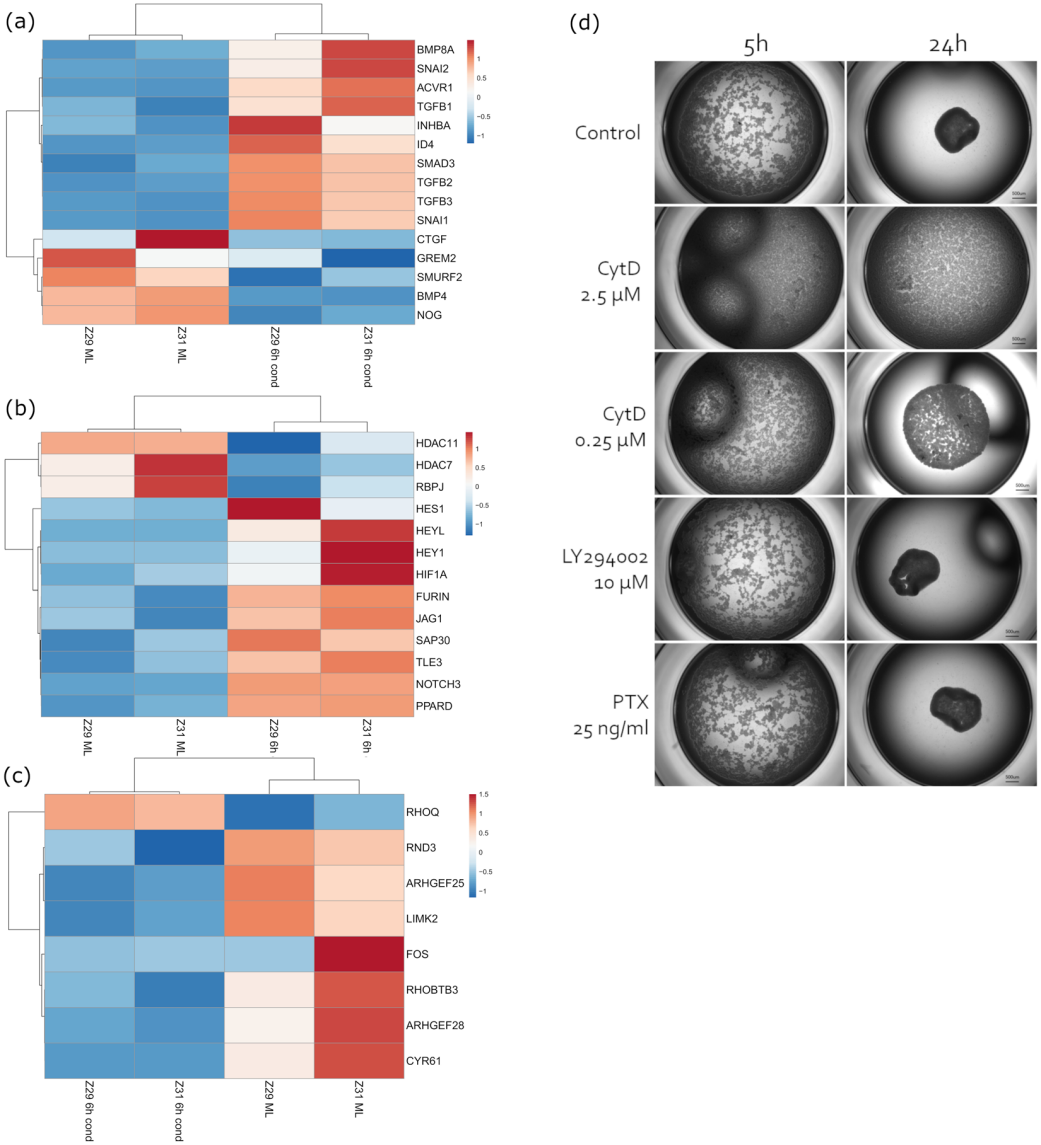
Clustered heatmap for selected gene panels. Significantly differentially expressed genes from DESeq2 analysis are depicted for (**a**) ‘*KEGG – TGFß signaling pathway*’ (**b**) ‘*Reactome – signaling by Notch*’ *and* (**c**) *a representative g*ene set generated by merging the gene panels ‘*PID RHOA REG PATHWAY*’ and ‘*PID RHOA PATHWAY*’ from the Pathway Interaction Database (PID, http://pid.nci.nih.gov) and embedding of RhoA activating GEFs (guanidine exchange factor) and inactivating GAPs (GTPase activating factor). (**d**) Inhibition of condensation by addressing different pathways via small molecules (see text for details). Pictures were taken 5 and 24 hours after condensation induction under influence of denoted molecules. Representative images (N = 2).

The small GTPase RhoA constitutes a link between TGFß mediated modulation of the actin cytoskeleton, two of the GO terms emerged in GSEA. To assess the impact of DPC condensation in the RHOA signal transduction pathway a representative gene set was generated by merging the gene panels PID RHOA REG PATHWAY and PID RHOA PATHWAY from the Pathway Interaction Database (PID, http://pid.nci.nih.gov) and the embedding of RhoA activating GEF (guanidine exchange factor) and inactivating GAP (GTPase activating factor) molecules resulting in 141 genes. Remarkably, the set of differentially expressed genes of the defined panel is characterized by downregulation of two Rho GTPases (*RND3* and *RHOBTB3*) as well as the transcriptional reduction of GTPase activating guanidine exchange factor molecules *ARHGEF25* and *ARHGEF28 (*Fig. 5c). The Lim kinase 2 (*LIMK2*) phosphorylates downstream targets in the Rho signal transduction pathway and is activated by the Rho/Rock. Significantly decreased RNA transcript levels for *LIMK2* enzyme and the downregulation of the direct Rho axis target gene *CYR61* emphasize the hypothesis of mitigated RhoA signaling activity.

Furthermore, advanced analysis independent of differential expression of genes demonstrates a broad expression of genes associated with the Rho pathway (Supplemental Fig. S3), verifying the general functionality of the pathway that can be modulated by the differentially expressed genes.

### Inhibition of condensation with small molecules

The impact of particular inhibitory molecules inspired by Mammoto *et al*.^16^ was determined to prove that our model of condensation depends on the remodeling of the actin cytoskeleton. Additionally, to an inhibitor of actin filament polymerization, cytochalasin D (2.5 μM or 0.25 μM), also an inhibitor of PI3K-Akt pathway (LY294002 at 10 μM), and an inhibitor of G*𝛼*i protein-coupled receptors (25 ng/ml), pertussis toxin (PTX), were administered into the culture medium prior to initiation of condensation in the ultra-low attachment wells. The inhibitors LY294002 and PTX had no visible effect on cell aggregation. The inhibitor of actin filament polymerization cytochalasin D adversely affected the condensation dose dependently (Fig. 5d). While 2.5 μM cytochalasin D entirely disrupted the process of condensation, a lower concentration of 0.25 μM, resulted in decreased cell-cell contacts and delayed condensation after 5 hours. After 24 hours, cells treated with a low concentration of the inhibitor condensed, but to a much smaller extent than the control cultures. To exclude impaired viability by cytochalasin D treatment, cells from all conditions were harvested and stained with the cell viability dye 7-aminoactinomycin D (7-AAD) (data not shown).

### Interaction of inductive condensates with epithelial cells

**Figure 6:**
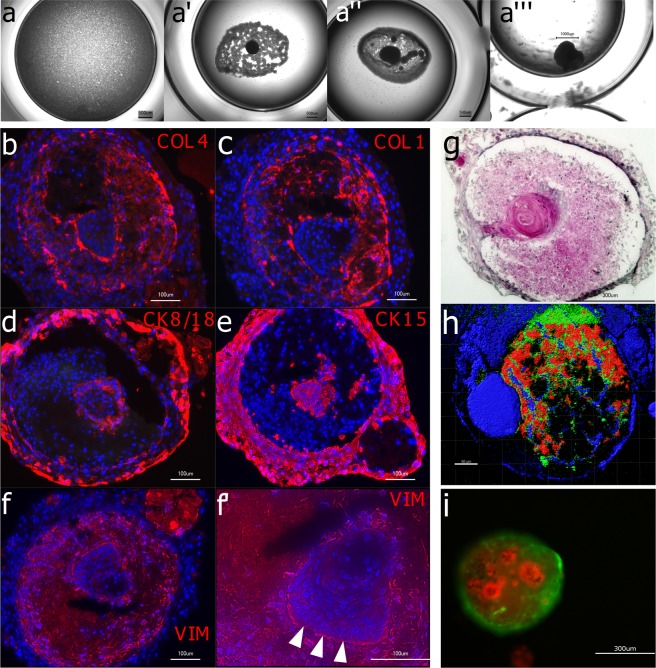
Interaction of inductive condensates with epithelial cells (**a**): Human gingival keratinocytes do not condensate *in vitro* in ultra-ow attachment plates when cultured alone. In co-culture with 24 h-old DPC condensates they accumulate around the DPC condensate (a’), enwrap it (a”) and after 4 weeks a dense co-culture aggregate has formed (a”’). Immunohistological analyses (**b**–f’) show a structured organization within the co-culture aggregate after 4 weeks. Collagen Type IV (**b**) and Collagen Type I (**c**) are located in the middle cell layer with a visible accumulation on the tissue borders. Epithelial-specific keratins such as Cytokeratin 8/18 (**d**) and Cytokeratin 15 (**e**) are located in the outer layer of the co-culture aggregate and in the inner core. The mesenchyme marker Vimentin (**f**) is expressed by the middle layer of cells. Note the columnar arrangement of the epithelial cells in the inner core on the epithelium-mesenchyme border (f’, arrow tips). Hematoxylin-Eosin staining (**g**) shows an epithelial-band-like structure indicating the migration of the keratinocyte into the mesenchymal condensate. In (**h**) a co-culture aggregate was analyzed in 2-photon microscopy without labelling and an excitation wavelength of λ = 1300 nm using the SHG effect (red channel) and autofluorescence (blue and green channel). In (**i**) the two interacting cell types were labelled and tracked under fluorescent microscopy. DPCs were transduced to express GFP (green) and gingival keratinocytes were labelled with CellTrackerRed (red). Also, here the self-assembly and tissue organization in the *in vitro* culture is evident.

*In vivo* the inductive mesenchyme gives rise to odontogenic cell fate in the dental and non-dental epithelium. To show that the produced condensate from adult dental pulp cells is indeed capable to induce interaction and invagination of epithelial cells, a co-culture was performed with keratinocytes from gingiva. After 24 hours of condensation, the dense DPC aggregates were co-cultured in ultra-low attachment wells with suspended adult oral keratinocytes. After additional 18 hours, the epithelial cells showed to have been attracted by the mesenchyme after 18 h of co-culture (Fig. 6a’), enwrapped it (Fig. 6a”) and formed densely packed aggregates consisting of the two cell types (Fig. 6a’”). The keratinocytes themselves did not condense in a mono 3D culture (Fig. 6a).

To distinguish between the two cell types in fluorescence microscopy, the dental pulp cells were transduced to express eGFP, and the epithelial keratinocytes were labeled with CellTracker Red. After four weeks of 3D culture, the two cell types were tightly grown together with a proportion of red keratinocyte inside the condensate, or, at least underneath the green DPCs reflecting the capability of invagination (Fig. 6i).

After four weeks of co-culture, the gingival epithelial cells formed a sheath around the mesenchymal cells and the oral keratinocytes invaginated inwards into the mesenchyme. Hematoxylin/Eosin overview staining (Fig. 6g) and imaging by 2-photon-microscopy (Fig. 6h) impressively demonstrate the stratified co-culture aggregate and the invaginated proportion of epithelial cells. From histological analysis by fluorescence staining with antibodies against either mesenchymal proportion (Collagen type I, Collagen type IV, Vimentin) or epithelial tissue (Cytokeratin 15, Cytokeratin 8/18) can be concluded that the epithelium not only lined the outer border but also formed a cluster of cells inside the mesenchymal condensate (Fig. 6b–f). The center of this cluster was acellular but rich in extracellular matrix, which was not composed of collagen type I or type IV as seen in the H&E overview and corresponding staining. The inner epithelial cells, which lined the border to the mesenchyme, arranged columnar, indicating the formation of an epithelial basal layer (see arrowhead Fig. 6f’ - high magnification of Vimentin staining).

## Discussion

The successful translation from bench to bedside of organoid technology-based application in regenerative medicine requires detailed knowledge about the organ development as well as an accessible source of cells the organoid is made of. However, present knowledge about tooth development is based on murine data or *in vitro* models exploiting embryonic murine cells. The present study represents the emulation of the initial step of *in vivo* tooth development, the process of mesenchymal condensation. Dental pulp cells isolated from wisdom teeth from adult donors were used for organoid formation. Adult stem cells from the dental pulp are ontogenetical derivates of the neural crest. The dental pulp and the dental pulp cells derive from the ecto-mesenchymal tissue, thus representing a mesenchymal phenotype arisen from ectoderm. The isolation of human dental pulp cells involves antigenic phenotyping and characterization. We show that the isolated cells in passage 1 express the surface markers for undifferentiated mesenchymal stromal cells (MSCs), and compliant with previous reports, have a comparably high proliferation rate^14,17,18^. They can reliably differentiate into adipogenic, osteogenic and neurogenic lineage by usage of specifically formulated differentiation media concordant with the findings of other groups^19,20^.

When primary cells from all tissue sources are isolated, they are dislodged from their natural three-dimensional environment consisting of neighboring cells and extracellular matrix. It is only logical that they change their expression profile, e.g. with respect to adhesion and signaling molecules as well as cell cycle genes and other important molecules. For many cell types (chondrocytes, hepatocytes, and more) this process is referred to as dedifferentiation of cells upon cell culture and is accompanied by loss of cell type-specific marker molecules and re-entering the cell cycle. Here, permissive culture conditions emulating the initial *in vivo* tooth differentiation process, the mesenchymal condensation, were applied to induce expression of odontogenic marker genes. At the site of presumptive tooth formation at around week 5 of human fetal development, the ecto-mesenchymal cells undergo mesenchymal condensation, forming the tooth bud. It is now accepted that after that process, the odontogenic fate lies in the mesenchymal condensate^7^. To mimic this condensation process, DPCs were cultivated in non-adherent conditions. After 12 hours all cells were integrated into a cellular network, and afterwards, the cell-cell-attachments led to an intense self-orchestrated aggregation. To estimate the differentiation upon 3D culture in comparison to *in vitro* expanded DPCs, the gene expression of 9 marker genes was measured. The majority of the target genes display downmodulated RNA levels in the amplification culture. Interestingly, upon 3D condensation of expanded DPCs, the phenotype can be rescued partially.

TGFß1 is described to be expressed by secretory odontoblasts as well as fibroblasts of the dental pulp^21^. TGFß1 is known to be a regulator of growth, cytodifferentiation, and inducer of matrix deposition. TGFß1 and TGFß2 regulate DSPP and DMP1 expression by odontoblasts as well as collagen expression by fibroblasts in the dental pulp^22,23^. This is in accordance with the congruent transcript expression profile by monolayer culture and condensation induced re-differentiation of the DPCs.

During early tooth development, the transcription factors PAX9 and MSX1 act synergistically to induce tooth initiation. The prime odontogenic potential lies in the dental epithelium. With the expression of FGFs and BMPs by the epithelium and subsequent induction of PAX9 and MSX1 in the mesenchyme, the odontogenic potential then shifts to the dental mesenchyme. In homozygous Pax9- as well as in Msx1-mutant mice the mesenchyme fails to condense normally^24^. In murine molar development, PAX9 is constantly expressed during all developmental stages of the dental mesenchyme. Before any morphological manifestation of tooth development, PAX9 is expressed at the prospective sites of tooth formation, but not in between the individual site for each tooth^25^. Interestingly, the expression of Pax9 was not affected by monolayer cultivation and is constantly expressed on a high level, demonstrating its central role in dental pulp cells. This finding, together with tooth agenesis upon Pax9 loss, concludes that PAX9 is a marker for dental mesenchyme which is needed to transduce the incoming epithelial signals to achieve tooth formation. It is therefore of particular interest that the PAX9 expression is not lost here during monolayer culture, suggesting that the used cells are capable to respond to appropriate signals with dental differentiation. The expression of MSX1 has been described to be unique for human dental pulp cells when compared to other cell types from various tissues^26^.

Furthermore, we compared the expression of these chosen marker genes to the expression of *in vitro* expanded mesenchymal stromal cells from bone marrow in passage 6 (MP6). Except for FGF-2, all genes were higher expressed in freshly isolated DPC compared to cultured bmMSC. The transcription factors MSX1 and PAX9 were barely detected or not expressed in bmMSC, respectively. This finding underlines the difference of mesenchymal progenitors from different sources even after *in vitro* cultivation defining particular capabilities of the individual populations for regenerative applications^27,28^.

Thus, a suitable 3D culture setup was established to allow production of singular dental mesenchymal condensates of 500 μm in diameter *in vitro* to resemble the human *in vivo* equivalent during organogenesis. In this culture model, a self-orchestrated condensation of the DPCs was allowed to induce the mechanotransductional forces on the cells which take place during condensational processes *in vivo*. Long-term culture of the condensates induces the induction of odontogenic genes within the condensate that were downregulated in monolayer culture.

Transcriptional comprehensive analysis of early phase of the condensation process revealed that the differentially expressed genes are enriched in pathways and biological functions connected to mesenchymal condensation *in vivo*.

Transcriptome analysis suggests a substantial shift of the balance to the TGFß/Activin Smad2/3 signaling pathway. All three TGFß ligands, as well as *INHBA* transcripts, are significantly upregulated after 6 hours of mesenchymal condensation. Being the only expressed isoform from the Inhibin/Activin dimers, it can be concluded that only Activin, not Inhibin, is expressed. Additionally, the Activin receptor type I is significantly upregulated. Activins play a role during early odontogenesis. Ferguson *et al*.^29^ describe Activin A as “an early mesenchymal signal” expressed in the condensing mesenchyme of the tooth germ, and mice lacking the *Inhba* gene exhibit a molar arrest at bud stage. The down regulation of TGß signaling inhibitory molecules emphasis this finding. In precartilage development, the mechanochemical principles of condensation depend on the regulation of matrix and adhesion molecules like integrins, collagen, fibronectin and tenascin by TGFß signaling^30^.

An interesting indication for active TGFß pathway is the observation that two direct TGFß-target genes, *SNAI1* and *SNAI2* (Supplemental Table S3), are both significantly upregulated during condensation. Very few publications show the expression of SNAI1/SNAI2 in mesenchymal condensates, e.g., in dermal condensates of developing murine hair follicles and during palatal development of the chick^31,32^.

The TGFß signaling pathway targets the signaling of the small GTPase RhoA. RhoA is abundantly expressed in condensing DPC, however RhoA functions as a molecular switch, cycling between an active GTP- and an inactive GDP-bound state, independent from the total RhoA abundance. The decreased expression of GTPase activating guanine nucleotide exchange factors (GEFs) together with the down regulation of RhoA signaling target molecules suggests a reduced activity. The inhibition of RhoA activity is also seen *in vivo* during mesenchymal condensation of murine tooth development^15^. RhoA regulates actin assembly and stress fiber formation, thus diminished RhoA activity is in concordance with the described mechanism of cytoskeletal transduction of mechanical cues during mesenchymal condensation and the requirements on the cytoskeleton to allow differentiation and ECM deposition. FGF2 is significantly upregulated in the DPCs in condensations. FGF2 has been reported to antagonize Rho activation, leading to loss of stress fibers and regulating the reorganization of cortical actin^33^. Inhibitory experiments by interfering actin polymerization and thereby the actin skeleton re-arrangement by Cytochalasin D entirely blocks cell-cell-attachment and condensation, emphasizing the important function of actin skeleton remodeling during the condensation.

An intriguing result from the GSEA is the Notch activation signature, characterized by elevated transcription of ligand molecules, receptor molecules as well as pathway target molecules in parallel with decreased expression of inhibitory molecules like histone acetylases. From developmental biology, it is known that Notch plays a role in mesenchymal condensates of skeletogenesis, where Notch maintains the mesenchymal precursors in an undifferentiated state and through lateral signaling to the neighboring cells the distinct developmental zones of long bone growth are established^34^. The function of Notch is also described for mesenchymal condensates in feather bud development and kidney^35,36^. Although Delta and Notch have been shown to be expressed in murine mesenchyme of developing molar teeth^37^, so far, no function has been assigned to this pathway during tooth development, neither in the animal model nor in humans. Since TGFß1 and BMP4 induced the expression of DLL1 and NOTCH3 in explant cultures of murine dental mesenchyme, a role in odontoblast differentiation was suggested^37^. Thus, the presented organoid culture model may serve as a useful tool to reveal the Notch function in tooth initiating mesenchymal condensation.

The retrieval of potential to induce invagination of epithelial cells, which is a crucial step in tooth organogenesis, is evidenced by co-culture experiments with gingival keratinocytes. These epithelial cells tightly bind to the surface of the DPC condensates forming a sheath. The epithelial cell guided the invagination into the mesenchymal condensate^38^. The invaginated epithelial cells exhibit a keratinization in the center of the organoid resembling skin or mucosa differentiation rather than tooth development which can be accounted to the used epithelial cell type (adult gingival keratinocytes). Further research e.g. with an ameloblast-like cell line, embryonic cells or *in vivo* studies are needed to prove odontogenic potential of the hereby described mesenchymal dental condensates.

The study presents an easy and practicable method to culture human DPCs in ultra-low attachment culture represents a suitable *in vitro* model to resemble embryonic mesenchymal condensation and therefore, and is a valuable tool to elucidate the mechanisms of molecular events without the use of animal models and embryonic cells. Furthermore, the application of human adult DPCs from wisdom teeth enables the development of a promising strategy for whole tooth regeneration.

## Materials and Methods

All methods and experimental protocols were carried out in accordance with the guidelines and regulations of the Ethics board of the Charité – Universitätsmedizin Berlin and was approved by the Ethics board approval**:** EA2/117/13. All isolations of cells from human tissues were carried out in accordance ethics board guidelines and regulations with informed written consent of the donating patients or, if subjects are under 18, from a parent and/or legal guardian.

### Isolation of human dental pulp cells from adult third molars

Extracted third molars were collected with patient’s informed consent from 18 to 30-year old adults from Charité Center for Dental, Oral, and Maxillary Medicine (Berlin, Germany). Teeth were kept in DMEM high glucose (PAA, Linz, Austria) containing 10% FCS (Biochrom, Germany) and 1% antibiotic-antimycotic solution (PAA, Linz, Austria) and stored at 4 °C for not longer than 24 hours. Human dental pulp cells are isolated under sterile conditions according to a modified protocol^13^. Briefly, extracted specimens were wiped with ethanol to reduce contamination risk. To open the pulpal cavity, teeth were cracked mechanically. Pulp tissue was removed with forceps and placed into a PBS containing petri dish. The tissue was minced and washed twice with PBS to remove debris and blood. Enzymatic tissue digestion was performed with a collagenase (3 mg/ml)/dispase II (4 mg/ml) enzyme mix in PBS for 1 h at 37 °C. After filtering through a 70 μm cell strainer and washing with PBS by centrifugation (400 × g for 5 min), the cell pellet is resuspended in DMEM with 10% FCS and antibiotics. Isolated dental pulp cells were cultivated and expanded in a humidified atmosphere at 37 °C, 5% vol/vol CO2 in standard tissue culture flasks (Greiner Bio-One GmbH, Germany) in DMEM high glucose (PAA) containing 10% FCS (Biochrom, Germany) and 1% antibiotic-antimycotic solution.

### Isolation of gingival keratinocytes

Residual gingival tissue from molar extractions (see above) was enzymatically digested with dispase II (4 mg/ml) for 30 min at 37 °C. After filtering through a 70 μm cell strainer and washing with PBS by centrifugation (400 × g for 5 min), the cell pellet is resuspended in complete DermaLife Basal medium supplemented with DermaLife K LifeFactors (Lifeline Cell Technology). Cell are grown on Collagen-A-coated (0,1 mg/mL Collagen A) tissue culture vessels under standard culture conditions.

### Isolation of human mesenchymal stromal cells from bone marrow

Human bmMSCs were isolated from femoral head marrow, obtained after joint replacement surgery, with written consent as per the guidelines of the Ethics board of the Charité - Universitätsmedizin Berlin, as previously described^39^. The cells were then expanded in DMEM high glucose (PAA) containing 10% FCS (Biochrom, Germany) and 1% antibiotic-antimycotic solution.

### Condensation process for formation of three-dimensional dental pulp constructs

For culture under non-adherent conditions, human dental pulp cells of passage 2–8 are passaged two days prior to use. They were harvested and resuspended in DMEM with 10% FCS to yield up in a single cell suspension of 10^6^ cells per ml. The cell suspension (0.2 ml per well) was given to 96 well low attachment plate (96-well Clear Flat Bottom Ultra-Low Attachment Microplate, Corning, Germany). In contrast to the negatively charged, hydrophilic surface of standard tissue culture dishes the ultra-low attachment plates possess a neutral, hydrophilic hydrogel coated surface that greatly minimizes the binding of attachment proteins. Condensation process starts shortly after seeding and was observed macroscopically by cells forming aggregates. To ensure constant culture conditions medium was changed regularly every 3 days. Cultures were kept under normal cell culture conditions. Small inhibitory molecules (Pertussis Toxin, Cytochalasin D and LY294002 (VWR)) were reconstituted according to supplier’s recommendations and administered to the culture wells with the DPCs in the designated concentrations. After five and 24 hours, microscopic pictures were taken.

### Co-culture assays

For co-culture of human dental pulp-derived cells and cells of epithelial origin (gingivium or skin derived) the condensates produced described by the method above are transferred at day 2 to 5 in a composite medium appropriate for both cell types (DMEM high glucose (with FCS)) and DermaLife Basal medium supplemented with DermaLife K LifeFactors (Lifeline Cell Technology); 1:1). A single cell suspension of epithelial cells in a ratio of 1:4 related to the initial cell number used for mesenchymal condensation was added and the resulting mixture was cultured under non-adherent conditions. To ensure constant culture conditions, medium was changed regularly every 1–3 days.

### Cell labeling

Keratinocytes were labeled fluorescently with CellTracker Red CMTPX Dye (ThermoFisher). Briefly, keratinocytes were trypsinized and resuspended in pre-warmed working solution, containing serum-free DermaLife medium and 5 μM of the dye (solubilized in DMSO). Cells were incubated at 37 °C for 45 minutes under gentle agitation. After washing and resuspension in fresh medium, the cells convert the permeable dye to a membrane impermanent label via glutathione-S-transferase. CellTracker Red is excitable at 577 nm and emits at 602 nm, which corresponds to the red channel in the fluorescence microscope. DPCs were labeled with eGFP by standard transfection with the reporter plasmid pLL3.7.

### Adipogenic differentiation

Cultured DPCs were grown until confluency before the differentiation was induced by exchanging the media to adipogenic media (standard DMEM supplemented with 10% FCS and 1% antibiotic-antimycotics, 0.1 mM L-ascorbic acid phosphate, 0.5 mM isobutylmethylxanthine, 0.5 μM hydrocortisone, 60 μM indomethacin and 10 μg/ml insulin). Media was changed three times per week for three weeks. To confirm adipogenic differentiation, cell cultures were stained for lipid vacuoles by Oil Red O and gene expression of lipid marker genes (FABP4, LPL) was assessed by RT-qPCR. Before staining, the cells were fixed in 10% formaldehyde for 30 minutes at room temperature and washed with PBS. Lipid droplets were stained with freshly filtered Oil Red O working solution (0.7% in propylene glycol). Excessive Oil Red O was carefully rinsed off with tap water. The extent of intracellular lipid vesicles was documented microscopically.

### Neurogenic differentiation

Neurogenic differentiation was performed according to the protocol of Miura *et al*.^40^. Briefly, culture plates were coated with 0.1% gelatin for 30 minutes and allowed to dry for at least two hours. Cultured DPCs were then seeded in a low density of 10 000 cells per cm2 in Neurobasal Plus Medium (Life Technologies) supplemented with 2% B27 Plus (Life Technologies), 250 µM L-Glutamin, 20 ng/ml EGF, 40 ng/ml bFGF and 1% antibiotic-antimycotics. Medium was changed every three days. To confirm neurogenic differentiation, mRNA expression of neuro-specific genes (Tuj1 and Nestin) was determined by RT-qPCR and cells were fixed in 4% PFA for 30 minutes at 4 °C. They were immunohistochemically stained for hNestin (eBioscience 14-9843-82) and hTuj1 (eBioscience 14-4510-82) and visualized with secondary antibody Goat anti-mouse CF 488 A (x3) (Biotium 20010).

### Osteogenic differentiation

DPCs were grown until confluency before osteogenic differentiation was induced. For this purpose, media was exchanged to “mineralization” media containing 10% FCS and 1% antibiotic-antimycotics, 0.1 mM L-ascorbic acid phosphate, 100 nM dexamethasone, 0.5 μM hydrocortisone, and 5 mM ß-glycerophosphate. Media was change regularly 2–3 times per week for three weeks. To confirm osteogenic differentiation, cell cultures were stained for mineralization (Alizarin red assay) and gene expression of osteogenic marker genes (OCN and OPN) was assessed by RT-qPCR. Before staining, the fragile cell layer was rinsed gently with PBS without calcium or magnesium, fixed in ice-cold 70% ethanol for one hour and washed twice with distilled water. Afterwards filtered Alizarin Red (2 g in 100 ml dH2O, pH 4.1 to 4.3 (HCl)) was incubated for 45 minutes at room temperature. After four washing steps with distilled water, the red staining of the mineralization was documented photographically.

### Flow cytometry

Cytometric analysis was carried out on MACSQuant Analyzer (Miltenyi Biotec, Germany). Cells in passage 1 were stained with fluorescently-labeled antibodies against surface markers CD105-APC (BioLegend, USA), CD106-APC (BioLegend, USA), CD90-FITC (Miltenyi Biotec, Germany), CD146-FITC (Miltenyi Biotec, Germany), CD13-APC-Cy7 (BioLegend, USA), CD44-PacificBlue (Miltenyi Biotec, Germany), CD45-VioBlue (Miltenyi Biotec, Germany), CD14-FITC (eBioscience, USA), CD34-APC (Miltenyi Biotec, Germany) and CD31-Alexa405 (DRFZ, Germany) for 10 minutes on ice followed by a washing step prior to analysis. Labeling of cells with CFSE was carried out using the CellTrace CFSE Cell Proliferation Kit (invitrogen, Thermo Fisher Scientific).

### RT-q-PCR

Isolation of RNA was performed by using Nucleospin RNA (MACHEREY-NAGEL) following the manufacturer’s protocol. Reverse transcription of mRNA (150 ng total RNA per reaction) was carried out by using TaqMan Reverse Transcription Reagents cDNA kit (Applied Biosystems) according to manufacturer’s protocol. Quantitative analysis of RNA expression by Real Time PCR was performed in the Stratagene MX3005P QPCR System (Agilent Technologies, Germany) by SYBR Green format using the SensiFAST SYBR No-ROX Kit (Bioline) by using 1 µl of cDNA and 0.25 µM primers (see Table S1). To exclude non-specificity of the PCR reaction due to contamination or primer dimers, melting curve analysis was performed after each PCR run. Gene expression levels were normalized to Glyceraldehyde 3-phosphate dehydrogenase (GAPDH).

### Whole transcriptome analysis (RNA-Seq)

Transcriptome analysis was performed by using next‐generation sequencing (NGS) from Illumina. First, a cDNA library was generated employing the TruSeq Stranded mRNA LT Sample Prep Kit (Illumina), according to the TruSeq Stranded mRNA Sample Preparation Protocol LS (Illumina). The initial quantity of total RNA was 1 µg for both monolayer DPCs and condensed cell. Diverse purification steps to separate the nucleic acid from reaction mix between the steps were performed with Mag‐Bind RXNPure Plus magnetic beads (Omega Bio‐tek). As a quality control and to determine the concentration and size of the fragments, the cDNA was analyzed with a UV‐Vis spectrophotometer (NanoDrop2000, Thermo Scientific) and gel electrophoresis (2% agarose). Purity of the generated mRNA-based library was verified by PCR for genomic DNA or ribosomal RNA fragments.

The sequencing was carried out by the Illumina MiSeq System. Raw data, generated by the Illumina MiSeq platform, were processed on the Galaxy Project platform^41^, using the tools FASTQ Groomer, to convert the output data into Sanger sequencing data. Fragments were then mapped against the human genome (hg38) to detect splice junctions between exons by HISAT2. Mapped reads were processed to the featureCounts tool on the Galaxy Project platform. Differential expression analysis was performed by the DESeq2 algorithm (nominal p-value < 0,05; FD > 2) provided by DE analysis (https://yanli.shinyapps.io/DEApp/, bioinformatics core, Center for Research Informatics (CRI), Biological Science Division (BSD), University of Chicago).

Differentially regulated genes were analyzed for overrepresented gene sets by GSEA (Gene Set Enrichment Analysis – http://software.broadinstitute.org/gsea/index.jsp; Broad Institute, Cambridge)^42^. Heatmap generation was conducted by ClustVis analysis platform^43^ and the Morpheus tool from Broad Institute Cambridge (https://software.broadinstitute.org/morpheus).

### Histology

Samples for histological analyses were embedded in cryomolds in O.C.T. Compound (Tissue-TEK) and frozen at −80 °C. Prior to sectioning by the cryotome (Leica), the frozen blocks were equilibrated at −20 °C for 30 minutes. At a cutting temperature of −18 °C 8 μm sections were transferred on glass slides (Histobond, Marienfeld, Germany). Slides were stored at −20 °C after two hours of drying at room temperature. Slides were thawed at room temperature and fixed in 10% formalin for 10 minutes at room temperature. After washing twice with distilled water, they were transferred to Hematoxylin solution and incubated for 5 minutes. Slides were then washed in running tap water for 10 minutes and cleared in distilled water. The slides were transferred into Eosin solution, incubated for 2 minutes and after washing with distilled water, they were dehydrated in a graded ethanol series with terminal xylene treatment. Prior to microscopic analysis, they were mounted in a resinous medium. After thawing, the slides were fixed in acetone for 20 minutes at −20 °C. Slides were washed three times in PBS. Unspecific binding was blocked with 10% serum from host animal of the secondary antibody for 20 minutes. Afterwards, primary antibody (Table S2) dilution was added to the sections and was incubated overnight at 4 °C. After washing with PBS, secondary antibody dilution was pipetted on the sections and was incubated for 45 minutes at room temperature in the dark. During the last 10 minutes of this incubation, DAPI (1:500) was added to the solution. After the last washing step, the sections were covered with Imsol Mount and a coverslip and were analyzed by fluorescence microscopy and 2-photon microscopy.

### Statistics

Mann-Whitney test was applied to the data sets, using GraphPad Prism software version 6.04 (GraphPad Software Inc., USA). P values smaller than or equal to 0.05 were considered significant.

## Supplementary information

Supplementary Information

cell condensation

## Data Availability

The authors state no restriction on data availability.
