# Supplementary material for: Emulating the early phases of human tooth development in vitro
Source: Sci Rep. 2019 May 7;9:7057. doi: 10.1038/s41598-019-43468-0 (PMC6505527; doi:10.1038/s41598-019-43468-0)
Supplement: Supplementary file 1 — Supplementary Information [file 41598_2019_43468_MOESM1_ESM.pdf]

Emulating the early phases of human tooth development *in vitro*

Supplementary information

Jennifer Rosowski[1], Julia Bräunig[1,2], Anna-Klara Amler[1,3], Frank P. Strietzel[4], Roland Lauster[1], Mark Rosowski[1,*]

[1] Technische Universität Berlin, Institute of Biotechnology, Department Medical Biotechnology, Gustav-Meyer-Allee 25, 13355 Berlin, DE

[2] Institute of Experimental Pediatric Endocrinology, Charité - Universitätsmedizin Berlin, Corporate Member of Freie Universität Berlin, Humboldt-Universität zu Berlin, and Berlin Institute of Health, Berlin, Germany.

[3] Cellbricks GmbH, Gustav-Meyer-Allee 25, 13355 Berlin, DE

[4] Charité · Charité Center for Dental, Oral, and Maxillary Medicine, Aßmannshauser Str.4-6 14197 Berlin, DE

*Corresponding author

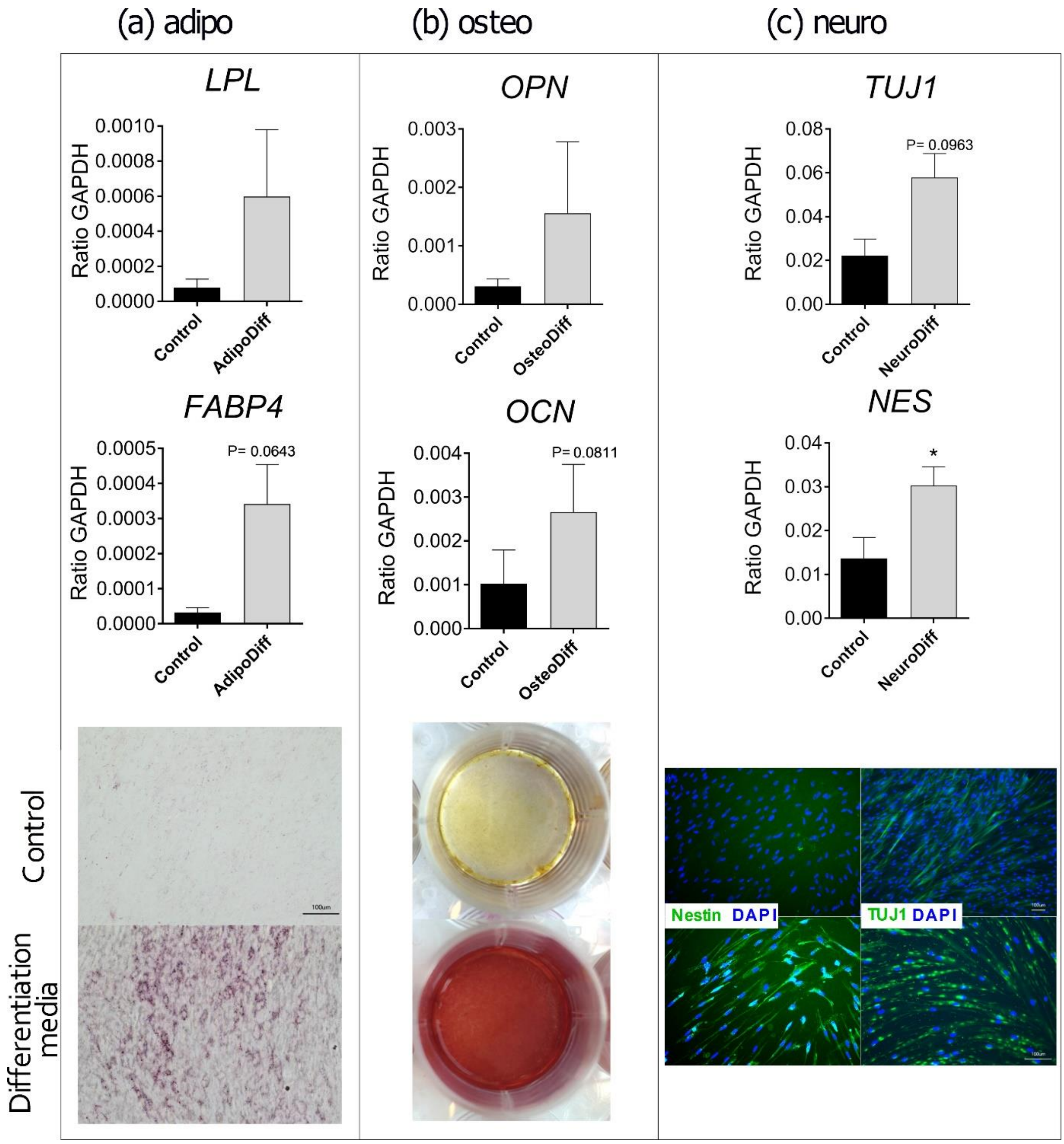

Figure S1 Multipotent differentiation capacity of isolated DPCs is shown on transcriptional level of selected marker genes for (a): adipogenic (FABP4, LPL), (b): osteogenic (OPN, OCN) and (c) neurogenic (NES, TUJ1) lineage relative to GAPDH expression. On the lower panel of a, b and c, differentiation was evidenced morphologically by staining of lipid vacuoles by Oil Red O for adipogenic differentiated DPCs, staining of calcification by Alizarin Red Assay for osteogenic differentiation and immunocytological staining of Nestin or Tuj1 for neurogenic differentiation. Control cells were cultured under the same condition but with standard DMEM supplemented with 10% FCS and P/S. (N=5 biological replicates, * P value < 0.05).

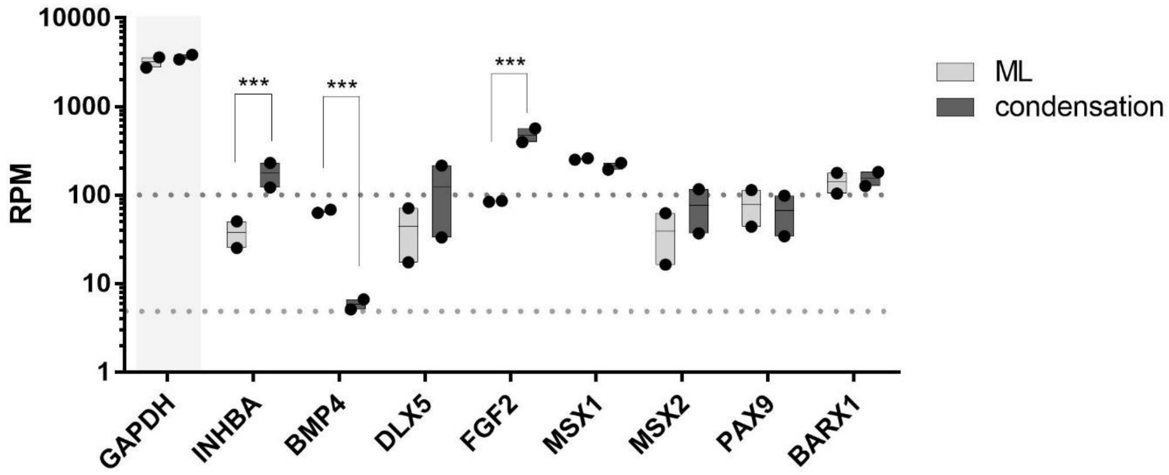

Figure S2 Gene expression of selected marker genes for initiation phase RPM values of tooth initiation associated genes are depicted. Dashed line marks the level of high expressed genes (RPM>100); dotted line marks the level of moderate expressed genes (RPM>5); [DESeq2 analysis: *** P value < 0.001; ** P value < 0.01; * P value < 0.05].

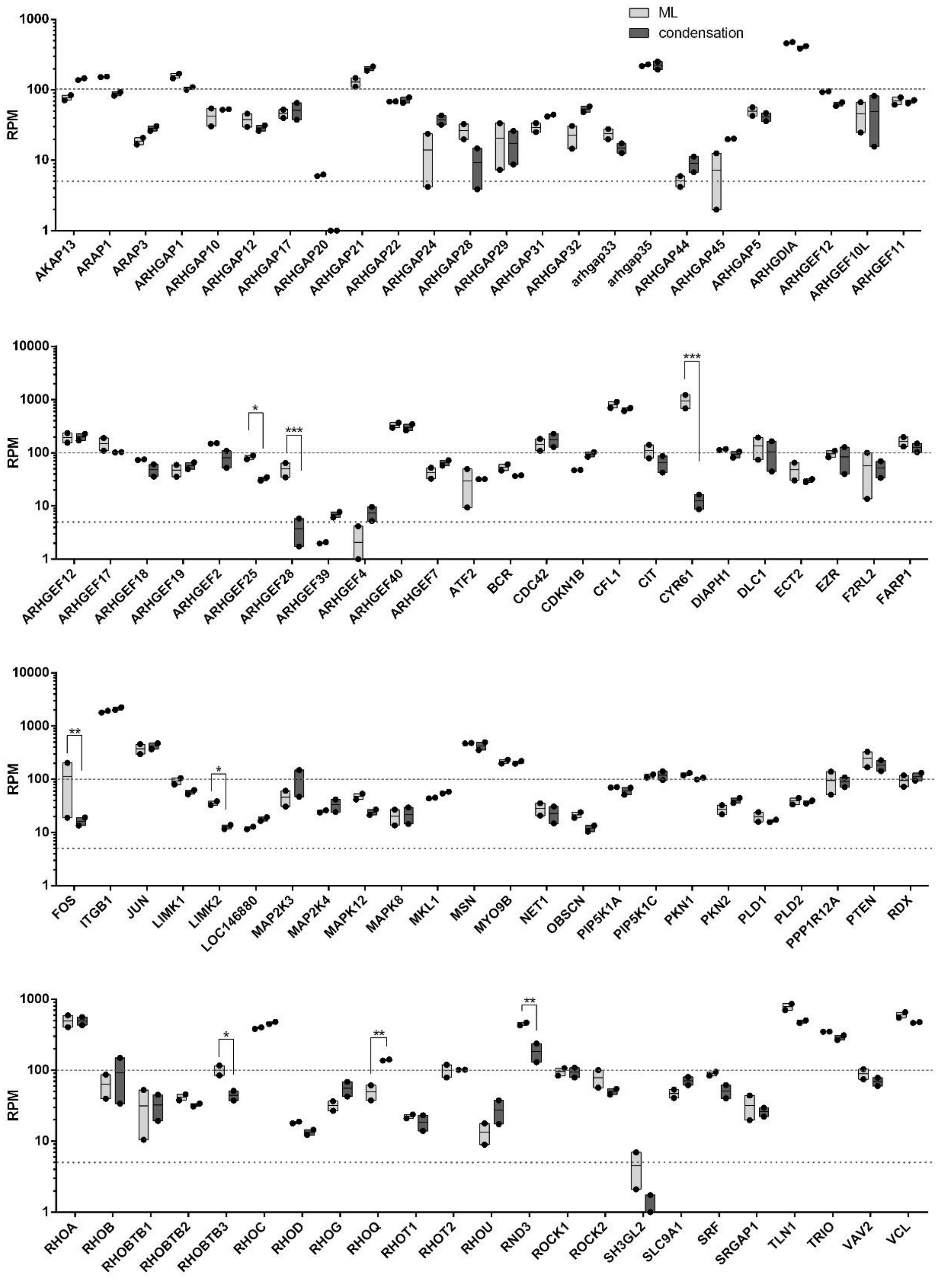

Figure S3. **RHOA signaling pathway** RPM values of RHOA transduction pathway associated genes are depicted (see text for panel composition). The diagram is reduced to genes with at least moderate gene expression level in the DPC condensation samples and the by genes with at least a two-fold difference in the RPM values of both samples [95 genes]. Dashed line marks the level of high expressed genes (RPM>100); dotted line marks the level of moderate expressed genes (RPM>5); [DESeq2 analysis: *** P value < 0.001; ** P value < 0.01; * P value < 0.05].

table S1. Primer pairs used for qPCR

GAPDH up	5'TGTTGCCATCAATGACCCCTT
GAPDH do	5'CTCCACGACGTACTCAGCG
BMP4 up	5'CGGGATCTTTACCGGCTTC
BMP4 do	5'TCTGCTGGGGGCTTCATAAC
BMP7 up	5'ACTGTGAGGGGGAGTGTGC
BMP7 do	5'CGAAGTAGAGGACGGAGATGG
INHBA up	5'GGGAGAACGGGTATGTGGAG
INHBA do	5'CTGGAAGAGGCGGATGGT
TGFß1 up	5'CCTGGCGATACCTCAGCAAC
TGFß1 do	5'GCCATGAGAAGCAGGAAAGG
FGF2 up	5'AGCGACCCTCACATCAAGC
FGF2 do	5'GCCCAGTTCGTTTCAGTGC
HGF up	5'GCCATGAATTTGACCTCTATGAAAAC
HGF do	5'TTTACCCCGATAGCTCGAAGG
PAX9 up	5'CCTACCACAgCCCCAAGGT
PAX9 do	5'AGCAACATAACCAGAAGGAGCAG
MSX1 up	5'GAGAGGACCCCGTGGATG
MSX1 do	5'CGATGGACAGGTACTGCTTCTG
DSPP up	5'GGCCATTCCAGTTCCTCAAA
DSPP do	5'TGGGTATTCTCTTGCCTTCCTC
COL1A1 up	5'GCCGTGACCTCAAGATGTG
COL1A1 do	5'GCCGAACCAGACATGCCTC
OPN up	5'CACTGATTTTCCCACGGACCT
OPN do	5'CCATTCAACTCCTCGCTTTCC
OCN up	5'CTCACACTCCTCGCCCTATTG
OCN do	5'CTCCCAGCCATTGATACAGGTAG
LPL up	5'GGCTGAAACTGGGCGAATCTAC
LPL do	5'CGTTGGAGGATGTGCTATTTGG
FABP4 up	5'GCAGAAATGGGATGGAAAATCA
FABP4 do	5'CGTCCCTTGGCTTATGCTCTC
Nestin up	5'AGGCTGAGGGACATCTTGAGG
Nestin do	5'AGCGTTGGAACAGAGGTTGG
TUJ1 up	5'CAGCAAGGTGCGTGAGGAG
TUJ1 do	5'TGCGGAAGCAGATGTCGTAG
SDC up	5'GGGGAGCAGGACTTCACCT
SDC do	5'TCCCAGCACCTCTTTCCTGT
TNC up	5'GGGACAGCAGGTGACTCCAT
TNC do	5'TGTCCCCATATCTCCCCATC
NCAM up	5'GTTCAAGCAACACCCCCTCTT
NCAM do	5'TTCTTCACCAACTGCTCTCCAC
FN1 up	5'CAGACCTATCCAAGCTCAAGTGG
FN1 do	5'TGGGTGGGATACTCACAGGTC

table S2. Antibodies for immunhistochemistry

Antibody	Distributor
mouse anti-human Collagen type I	Sigma C2456
mouse anti-human Collagen type IV	Sigma C1926
mouse anti-human Cytokeratin8/18	Invitrogen MA5-14088
rabbit anti-human Cytokeratin 15	Abcam ab52816
rabbit anti-human Vimentin	Invitrogen PA5-27231

table S3. Differentially expressed genes between human DPCs in monolayer (ML) or six hours in condensation (6h) according to DESeq2 algorithm (|FC| ≥ 1.5, nominal P value< 0.05). Two biological replicates were performed. Upregulated genes are highlighted in red and downregulated in blue.

		Reads per Million (RPM)						
GeneID	Official Gene Symbol	ZZ29 ML	ZZ29 6h Cond	ZZ31 ML	ZZ31 6h Cond	log2 FoldChange	P value	FDR adj. P-value
7043	TGFB3	12,578	397,953	0,000	330,028	3,891	9,00E-17	3,08E-13
2847	MCHR1	0,000	80,365	0,000	69,663	3,354	1,46E-11	3,00E-08
1909	EDNRA	4,193	110,381	0,000	82,725	3,241	2,41E-11	4,13E-08
50509	COL5A3	129,977	1572,446	97,270	1565,674	3,184	1,03E-17	5,28E-14
4071	TM4SF1	28,301	506,397	38,709	459,775	3,183	2,12E-16	5,45E-13
10221	TRIB1	2,096	71,651	5,955	102,753	2,909	1,32E-09	1,69E-06
57801	HES4	2,096	50,349	2,978	82,725	2,836	9,45E-09	9,71E-06
54498	SMOX	102,723	1222,906	45,657	560,787	2,670	2,56E-09	2,92E-06
4773	NFATC2	2,096	54,222	0,000	40,927	2,647	1,94E-07	9,80E-05
3400	ID4	29,350	336,953	34,739	235,112	2,541	4,71E-10	6,92E-07
3696	ITGB8	9,434	63,905	7,940	146,292	2,480	1,37E-07	7,80E-05
84632	AFAP1L2	8,386	87,143	9,925	103,624	2,462	5,59E-08	4,10E-05
4741	NEFM	11,530	152,016	3,970	62,697	2,453	5,48E-07	2,45E-04
3720	JARID2	18,868	151,048	13,896	141,938	2,427	1,69E-08	1,45E-05
57561	ARRDC3	16,771	172,349	34,739	271,685	2,417	1,46E-08	1,36E-05
8877	SPHK1	44,024	444,429	27,791	203,764	2,393	6,83E-08	4,68E-05
112399	EGLN3	0,000	20,333	1,985	69,663	2,374	4,66E-06	1,84E-03
5139	PDE3A	2,096	61,000	0,000	21,770	2,313	8,23E-06	2,68E-03
84959	UBASH3B	15,723	134,587	14,888	108,848	2,273	2,00E-07	9,80E-05
51129	ANGPTL4	46,121	509,302	8,933	148,034	2,240	7,20E-06	2,55E-03
6542	SLC7A2	10,482	45,508	11,911	191,573	2,211	6,05E-06	2,22E-03
56895	AGPAT4	25,157	119,095	12,903	168,933	2,174	9,14E-07	3,91E-04
340075	ARSI	0,000	48,413	3,970	29,607	2,164	2,49E-05	6,40E-03
57471	ERMN	2,096	26,143	0,000	37,444	2,162	3,01E-05	7,53E-03
6303	SAT1	71,277	344,699	68,486	502,444	2,144	2,19E-08	1,73E-05
100499467	LINC00673	8,386	82,302	18,858	122,781	2,127	3,06E-06	1,26E-03
84935	MEDAG	2,096	118,127	0,000	6,966	2,119	5,23E-05	1,12E-02
2247	FGF2	85,952	574,175	87,344	402,303	2,063	1,06E-07	6,71E-05
8835	SOCS2	31,446	168,476	10,918	136,713	2,053	7,52E-06	2,58E-03
9077	DIRAS3	17,819	114,254	1,985	80,983	2,040	4,43E-05	9,90E-03
23551	RASD2	0,000	6,778	1,985	94,916	2,039	9,95E-05	1,89E-02
26207	PITPNC1	63,940	316,619	58,560	350,927	2,025	1,11E-07	6,71E-05
9945	GFPT2	13,627	138,460	22,829	90,562	1,991	8,55E-06	2,68E-03
196740	VSTM4	67,085	367,937	33,747	233,371	1,969	5,71E-06	2,17E-03
643837	LINC01128	12,578	61,000	11,911	100,140	1,951	2,20E-05	5,81E-03
55326	AGPAT5	6,289	34,857	10,918	104,494	1,941	8,12E-05	1,66E-02
3754	KCNF1	4,193	29,048	3,970	47,022	1,910	1,58E-04	2,80E-02
84627	ZNF469	54,506	272,080	25,806	177,640	1,871	2,20E-05	5,81E-03
6615	SNAI1	10,482	66,810	8,933	54,860	1,845	9,32E-05	1,84E-02
392617	ELFN1	11,530	104,572	3,970	29,607	1,845	2,87E-04	4,46E-02
26508	HEYL	0,000	14,524	0,000	28,736	1,819	5,17E-04	6,72E-02
5376	PMP22	25,157	170,413	27,791	97,528	1,787	4,66E-05	1,02E-02
25861	WHRN	19,916	95,857	4,963	67,051	1,779	2,68E-04	4,38E-02
29923	HILPDA	7,337	52,286	10,918	55,730	1,768	2,06E-04	3,41E-02
1407	CRY1	6,289	48,413	7,940	41,798	1,755	3,30E-04	4,91E-02
3280	HES1	4,193	50,349	1,985	15,674	1,749	7,83E-04	9,46E-02
19	ABCA1	50,314	196,556	49,627	236,854	1,724	1,12E-05	3,19E-03
79870	BAALC	30,398	158,794	53,598	207,247	1,695	3,92E-05	9,15E-03
353500	BMP8A	2,096	21,302	4,963	38,315	1,688	1,07E-03	1,17E-01
10171	RCL1	6,289	16,460	2,978	64,438	1,684	1,12E-03	1,20E-01
83716	CRISPLD2	46,121	296,286	13,896	72,275	1,663	8,54E-04	9,74E-02

26353	HSPB8	2,096	12,587	3,970	47,893	1,653	1,54E-03	1,52E-01
50700	RDH8	0,000	17,429	0,000	17,416	1,636	1,78E-03	1,66E-01
3624	INHBA	51,362	234,318	25,806	123,652	1,632	3,06E-04	4,69E-02
220323	OAF	111,109	293,381	51,613	419,719	1,630	1,26E-04	2,27E-02
54541	DDIT4	201,254	888,858	72,456	409,270	1,621	4,27E-04	5,93E-02
182	JAG1	34,591	100,698	9,925	120,169	1,593	7,37E-04	9,12E-02
3554	IL1R1	274,628	1098,001	386,102	1311,405	1,592	8,87E-06	2,68E-03
5054	SERPINE1	801,872	2897,987	704,710	2586,236	1,583	9,80E-06	2,88E-03
9419	CRIPT	0,000	19,365	1,985	17,416	1,577	2,61E-03	2,12E-01
169792	GLIS3	50,314	175,254	8,933	137,584	1,574	1,25E-03	1,31E-01
3638	INSIG1	17,819	139,429	17,866	40,056	1,571	1,28E-03	1,33E-01
387763	C11ORF96	76,518	235,286	54,590	269,073	1,564	8,26E-05	1,66E-02
1942	EFNA1	0,000	7,746	1,985	35,702	1,559	2,89E-03	2,23E-01
145581	LRFN5	0,000	7,746	1,985	35,702	1,559	2,89E-03	2,23E-01
6932	TCF7	37,735	93,921	30,769	194,185	1,558	3,58E-04	5,18E-02
8553	BHLHE40	57,651	301,127	63,523	168,933	1,555	1,97E-04	3,33E-02
166647	adgra3	50,314	161,699	48,635	215,084	1,553	9,85E-05	1,89E-02
1846	DUSP4	9,434	68,746	3,970	19,157	1,540	2,78E-03	2,22E-01
284018	C17orf58	5,241	38,730	9,925	40,056	1,539	1,84E-03	1,66E-01
4216	MAP3K4	31,446	125,873	23,821	96,657	1,536	4,03E-04	5,75E-02
3775	KCNK1	5,241	25,175	3,970	32,219	1,526	2,83E-03	2,22E-01
2012	EMP1	462,255	2153,399	515,133	1311,405	1,523	7,24E-05	1,52E-02
7223	TRPC4	0,000	21,302	5,955	30,478	1,514	3,62E-03	2,62E-01
7779	SLC30A1	44,024	164,603	27,791	118,427	1,513	4,74E-04	6,40E-02
5163	PDK1	18,868	53,254	24,814	134,972	1,503	1,16E-03	1,23E-01
8013	NR4A3	13,627	74,556	1,985	26,994	1,498	3,73E-03	2,64E-01
83786	FRMD8	68,133	337,921	64,516	162,837	1,496	4,82E-04	6,40E-02
26035	GLCE	19,916	61,000	38,709	195,927	1,495	1,49E-03	1,49E-01
7378	UPP1	11,530	62,937	3,970	25,253	1,483	3,61E-03	2,62E-01
9052	GPRC5A	0,000	11,619	0,000	19,157	1,483	4,48E-03	2,90E-01
114088	TRIM9	0,000	13,556	0,000	16,545	1,479	4,60E-03	2,95E-01
136	ADORA2B	3,145	16,460	0,000	21,770	1,464	5,19E-03	3,15E-01
467	ATF3	9,434	45,508	3,970	26,994	1,460	4,01E-03	2,75E-01
56937	PMEPA1	49,265	218,826	69,478	187,219	1,448	2,77E-04	4,44E-02
6424	SFRP4	11,530	50,349	15,881	60,955	1,440	2,00E-03	1,76E-01
5142	PDE4B	35,639	107,476	9,925	87,949	1,427	2,82E-03	2,22E-01
10184	LHFPL2	47,169	152,016	68,486	244,691	1,420	4,86E-04	6,40E-02
6383	SDC2	138,362	312,746	167,741	761,067	1,419	5,99E-04	7,65E-02
85463	ZC3H12C	28,301	105,540	17,866	70,534	1,414	1,85E-03	1,66E-01
10579	TACC2	13,627	61,000	7,940	34,831	1,413	3,99E-03	2,75E-01
54852	PAQR5	10,482	38,730	22,829	102,753	1,410	3,50E-03	2,59E-01
23313	KIAA0930	109,013	259,492	77,419	366,601	1,399	4,12E-04	5,79E-02
6095	RORA	24,109	90,048	19,851	70,534	1,394	1,78E-03	1,66E-01
10560	SLC19A2	2,096	16,460	2,978	20,899	1,392	7,80E-03	4,20E-01
29116	MYLIP	8,386	46,476	17,866	57,472	1,379	3,79E-03	2,67E-01
94031	HTRA3	95,386	194,619	98,262	470,225	1,376	1,04E-03	1,16E-01
23401	FRAT2	3,145	28,079	0,000	9,579	1,369	8,91E-03	4,53E-01
23413	NCS1	85,952	295,318	110,173	313,483	1,364	2,85E-04	4,46E-02
4291	MLF1	0,000	21,302	5,955	22,640	1,351	9,69E-03	4,76E-01
7803	PTP4A1	55,555	195,587	61,538	174,157	1,351	6,29E-04	7,88E-02
23254	KAZN	16,771	40,667	10,918	67,921	1,346	4,71E-03	2,97E-01
6446	SGK1	36,687	133,619	23,821	79,242	1,345	2,77E-03	2,22E-01
406	ARNTL	4,193	39,698	9,925	25,253	1,344	8,06E-03	4,27E-01
80018	NAA25	4,193	43,571	16,873	43,539	1,323	7,87E-03	4,21E-01
2903	GRIN2A	0,000	24,206	0,000	5,225	1,323	1,04E-02	4,93E-01
687	KLF9	41,928	115,222	26,799	111,461	1,314	2,27E-03	1,93E-01
9586	CREB5	4,193	22,270	1,985	16,545	1,312	1,21E-02	5,31E-01
10570	DPYSL4	16,771	50,349	27,791	106,236	1,301	4,83E-03	2,99E-01
23704	KCNE4	6,289	57,127	0,000	5,225	1,300	1,28E-02	5,39E-01

2114	ETS2	41,928	143,302	6,948	67,051	1,299	9,76E-03	4,77E-01
153478	PLEKHG4B	0,000	22,270	7,940	26,994	1,298	1,28E-02	5,39E-01
51312	SLC25A37	81,759	241,095	45,657	168,933	1,295	2,46E-03	2,07E-01
55	ACPP	2,096	13,556	0,000	14,803	1,293	1,33E-02	5,49E-01
92104	TTC30A	2,096	13,556	5,955	32,219	1,288	1,36E-02	5,59E-01
7090	TLE3	26,205	99,730	42,680	114,944	1,280	2,58E-03	2,12E-01
57464	STRIP2	4,193	21,302	3,970	20,899	1,278	1,38E-02	5,64E-01
89797	NAV2	14,675	85,206	46,650	120,169	1,277	5,51E-03	3,26E-01
11138	TBC1D8	40,880	117,159	15,881	80,983	1,271	6,41E-03	3,62E-01
10808	HSPH1	81,759	288,540	76,426	184,607	1,270	1,79E-03	1,66E-01
85450	ITPRIP	88,049	336,953	67,493	152,388	1,267	3,70E-03	2,64E-01
6591	SNAI2	67,085	154,921	63,523	236,854	1,265	1,56E-03	1,52E-01
23462	HEY1	0,000	5,810	0,000	20,899	1,260	1,45E-02	5,73E-01
64131	XYLT1	95,386	297,254	112,158	290,843	1,253	8,47E-04	9,74E-02
84870	RSPO3	2,096	30,984	0,000	3,483	1,246	1,58E-02	6,00E-01
5159	PDGFRB	569,172	1950,065	515,133	1146,826	1,245	1,43E-03	1,45E-01
10758	TRAF3IP2	25,157	94,889	9,925	37,444	1,239	1,22E-02	5,33E-01
51010	EXOSC3	6,289	21,302	0,000	20,899	1,237	1,81E-02	6,44E-01
7042	TGFB2	100,627	308,873	109,180	278,652	1,235	1,08E-03	1,17E-01
4783	NFIL3	37,735	104,572	11,911	70,534	1,234	9,95E-03	4,84E-01
1956	EGFR	78,615	245,937	45,657	138,455	1,233	4,88E-03	3,00E-01
24145	PANX1	124,736	377,619	101,240	269,944	1,232	1,86E-03	1,66E-01
10397	NDRG1	198,109	595,477	121,091	350,927	1,228	3,33E-03	2,51E-01
2774	GNAL	2,096	5,810	0,000	24,382	1,226	1,82E-02	6,44E-01
23186	RCOR1	25,157	62,937	27,791	101,882	1,225	5,49E-03	3,26E-01
3717	JAK2	8,386	40,667	23,821	71,405	1,224	1,13E-02	5,17E-01
2300	FOXL1	6,289	24,206	5,955	26,994	1,221	1,66E-02	6,13E-01
6515	SLC2A3	25,157	119,095	44,665	89,691	1,221	5,06E-03	3,09E-01
57214	CEMIP	271,483	908,223	98,262	324,803	1,219	9,64E-03	4,76E-01
84803	GPAT3	0,000	14,524	0,000	8,708	1,218	1,82E-02	6,44E-01
55647	RAB20	0,000	13,556	1,985	12,191	1,218	1,94E-02	6,63E-01
1832	DSP	3,145	25,175	11,911	36,573	1,214	1,75E-02	6,28E-01
64651	CSRNP1	12,578	65,841	18,858	38,315	1,207	1,14E-02	5,19E-01
25799	ZNF324	4,193	19,365	0,993	13,933	1,204	2,15E-02	7,03E-01
23446	SLC44A1	123,687	259,492	116,128	411,882	1,203	1,86E-03	1,66E-01
114880	OSBPL6	2,096	22,270	7,940	23,511	1,198	2,10E-02	6,96E-01
85027	SMIM3	34,591	138,460	44,665	91,433	1,197	5,23E-03	3,16E-01
55893	ZNF395	85,952	177,191	101,240	357,893	1,194	3,40E-03	2,53E-01
6533	SLC6A6	107,964	329,207	47,642	155,000	1,189	9,19E-03	4,61E-01
3570	IL6R	11,530	53,254	2,978	14,803	1,188	2,24E-02	7,06E-01
23433	RHOQ	37,735	141,365	61,538	136,713	1,188	3,91E-03	2,73E-01
4163	MCC	49,265	129,746	38,709	121,910	1,188	4,25E-03	2,82E-01
57509	MTUS1	0,000	12,587	0,000	9,579	1,185	2,14E-02	NA
9700	ESPL1	12,578	36,794	10,918	42,669	1,184	1,42E-02	5,70E-01
7539	ZFP37	2,096	13,556	1,985	13,933	1,179	2,41E-02	7,43E-01
6197	RPS6KA3	51,362	142,333	87,344	246,433	1,171	4,77E-03	2,97E-01
864	RUNX3	56,603	169,445	48,635	124,522	1,170	4,77E-03	2,97E-01
201501	ZBTB7C	0,000	15,492	0,000	6,966	1,169	2,28E-02	NA
57596	BEGAIN	0,000	10,651	1,985	13,933	1,168	2,46E-02	7,44E-01
481	ATP1B1	8,386	23,238	8,933	39,185	1,154	2,11E-02	6,96E-01
2043	EPHA4	10,482	29,048	25,806	95,787	1,154	1,95E-02	6,64E-01
8609	KLF7	2,096	23,238	8,933	23,511	1,151	2,66E-02	7,80E-01
9695	EDEM1	63,940	124,905	66,501	236,854	1,147	5,53E-03	3,26E-01
285958	SNHG15	0,000	11,619	0,000	9,579	1,146	2,56E-02	NA
80853	KDM7A	4,193	23,238	11,911	36,573	1,143	2,46E-02	7,44E-01
8531	YBX3	94,338	263,365	101,240	246,433	1,143	2,59E-03	2,12E-01
9394	HS6ST1	104,820	287,572	61,538	177,640	1,140	7,68E-03	4,15E-01
6376	CX3CL1	2,096	29,048	1,985	3,483	1,139	2,77E-02	7,93E-01
9261	MAPKAPK2	187,627	461,858	159,800	438,006	1,138	2,24E-03	1,92E-01

9781	RNF144A	14,675	68,746	62,531	175,899	1,134	1,92E-02	6,63E-01
114299	PALM2	2,096	16,460	3,970	14,803	1,128	3,13E-02	8,38E-01
196527	ANO6	76,518	267,238	129,031	261,236	1,122	4,10E-03	2,76E-01
57132	CHMP1B	169,808	457,016	74,441	242,079	1,121	1,21E-02	5,31E-01
55818	KDM3A	18,868	72,619	52,605	134,101	1,116	1,53E-02	5,89E-01
55197	RPRD1A	19,916	52,286	14,888	51,376	1,116	1,65E-02	6,10E-01
285203	EOGT	29,350	49,381	28,784	121,910	1,112	1,53E-02	5,89E-01
221830	TWISTNB	20,964	62,937	22,829	60,955	1,111	1,33E-02	5,49E-01
171546	SPTSSA	39,832	86,175	29,776	105,365	1,111	9,62E-03	4,76E-01
160760	PPTC7	28,301	122,000	41,687	70,534	1,109	1,26E-02	5,39E-01
6792	CDKL5	0,000	4,841	0,993	17,416	1,105	3,02E-02	NA
283209	PGM2L1	14,675	26,143	11,911	59,213	1,093	2,58E-02	7,65E-01
81792	ADAMTS12	8,386	38,730	13,896	31,348	1,092	2,63E-02	7,74E-01
10123	ARL4C	81,759	297,254	22,829	59,213	1,092	3,06E-02	8,28E-01
54910	SEMA4C	63,940	239,159	57,568	94,045	1,091	1,52E-02	5,89E-01
6659	SOX4	100,627	209,143	231,264	736,685	1,091	1,75E-02	6,28E-01
51582	AZIN1	134,169	372,778	226,301	529,438	1,090	4,69E-03	2,97E-01
6482	ST3GAL1	88,049	254,651	72,456	161,096	1,088	8,38E-03	4,35E-01
3589	IL11	33,542	476,381	13,896	60,955	1,086	3,05E-02	8,28E-01
8808	IL1RL2	0,000	9,683	3,970	19,157	1,086	3,78E-02	9,18E-01
56925	LXN	12,578	23,238	26,799	107,978	1,085	3,03E-02	8,28E-01
84365	NIFK	2,096	28,079	10,918	21,770	1,085	3,65E-02	9,10E-01
157506	RDH10	109,013	289,508	295,780	829,860	1,080	1,71E-02	6,26E-01
10788	IQGAP2	0,000	6,778	1,985	16,545	1,079	3,66E-02	9,10E-01
50640	PNPLA8	16,771	62,937	24,814	52,247	1,076	1,88E-02	6,56E-01
83641	FAM107B	41,928	161,699	63,523	108,848	1,074	1,10E-02	5,09E-01
3566	IL4R	102,723	259,492	44,665	148,034	1,073	1,72E-02	6,27E-01
4007	PRICKLE3	5,241	24,206	4,963	15,674	1,071	3,93E-02	9,33E-01
57181	SLC39A10	2,096	19,365	13,896	40,927	1,068	3,93E-02	9,33E-01
1195	CLK1	20,964	67,778	40,695	97,528	1,067	1,65E-02	6,10E-01
2150	F2RL1	4,193	23,238	35,732	125,393	1,059	4,11E-02	9,45E-01
90627	STARD13	73,374	196,556	104,218	237,725	1,057	6,35E-03	3,62E-01
2013	EMP2	59,747	130,714	63,523	177,640	1,057	8,15E-03	4,27E-01
79656	BEND5	0,000	5,810	1,985	17,416	1,056	4,02E-02	9,38E-01
3755	KCNG1	14,675	39,698	21,836	62,697	1,051	2,47E-02	7,46E-01
57647	DHX37	30,398	69,714	18,858	64,438	1,048	2,10E-02	6,96E-01
93145	OLFM2	57,651	97,794	18,858	120,169	1,047	2,45E-02	7,44E-01
7074	TIAM1	4,193	16,460	13,896	47,022	1,044	4,25E-02	9,52E-01
121642	ALKBH2	4,193	11,619	1,985	18,287	1,043	4,66E-02	1,00E+00
5641	LGMN	30,398	61,968	25,806	86,208	1,040	1,84E-02	6,51E-01
6781	STC1	270,435	1834,843	38,709	792,416	1,039	4,08E-02	9,43E-01
10194	TSHZ1	24,109	89,079	28,784	51,376	1,039	2,27E-02	7,09E-01
116987	AGAP1	63,940	161,699	47,642	119,298	1,038	1,33E-02	5,49E-01
100133941	CD24	4,193	1,937	8,933	69,663	1,037	4,70E-02	1,00E+00
10435	CDC42EP2	12,578	51,318	1,985	11,320	1,036	4,78E-02	1,00E+00
6018	RLF	12,578	27,111	13,896	51,376	1,036	3,34E-02	8,66E-01
254170	FBXO33	15,723	48,413	12,903	33,961	1,036	3,16E-02	8,42E-01
30811	HUNK	6,289	37,762	9,925	16,545	1,035	4,34E-02	9,65E-01
55422	ZNF331	13,627	44,540	12,903	32,219	1,034	3,27E-02	8,58E-01
81831	NETO2	13,627	11,619	11,911	80,112	1,033	4,50E-02	9,85E-01
54793	KCTD9	36,687	129,746	54,590	96,657	1,030	1,50E-02	5,86E-01
56180	MOSPD1	2,096	8,714	3,970	20,899	1,028	4,95E-02	1,00E+00
23268	DNMBP	41,928	122,000	31,762	68,792	1,022	2,23E-02	7,06E-01
6461	SHB	44,024	101,667	33,747	94,045	1,022	1,65E-02	6,10E-01
3269	HRH1	14,675	34,857	3,970	27,865	1,021	4,56E-02	9,93E-01
153443	SRFBP1	2,096	13,556	0,000	7,837	1,020	4,77E-02	NA
3099	HK2	63,940	225,603	68,486	105,365	1,020	1,91E-02	6,63E-01
164284	APCDD1L	56,603	112,318	15,881	93,174	1,018	3,23E-02	8,50E-01
10116	FEM1B	51,362	108,445	58,560	160,225	1,017	1,27E-02	5,39E-01

83930	STARD3NL	20,964	64,873	31,762	69,663	1,015	2,23E-02	7,06E-01
5128	CDK17	16,771	65,841	30,769	57,472	1,014	2,63E-02	7,74E-01
10746	MAP3K2	26,205	59,064	24,814	72,275	1,013	2,25E-02	7,06E-01
665	BNIP3L	150,941	395,048	241,189	518,118	1,012	7,42E-03	4,04E-01
8819	SAP30	9,434	38,730	15,881	33,090	1,009	3,86E-02	9,28E-01
2034	EPAS1	420,327	1016,668	188,584	534,663	1,007	2,20E-02	7,06E-01
57597	BAHCC1	98,531	267,238	108,188	215,955	1,007	9,07E-03	4,57E-01
8497	PPFIA4	12,578	17,429	12,903	62,697	1,007	4,56E-02	9,93E-01
22856	CHSY1	66,036	188,810	85,359	167,191	1,006	1,06E-02	4,98E-01
23132	RAD54L2	16,771	67,778	30,769	54,860	1,005	2,81E-02	7,95E-01
79720	VPS37B	44,024	116,191	48,635	106,236	0,999	1,53E-02	5,89E-01
5045	FURIN	227,459	445,397	167,741	479,803	0,999	8,28E-03	4,32E-01
1130	LYST	17,819	60,032	31,762	66,180	0,998	2,71E-02	7,84E-01
4088	SMAD3	146,748	389,238	180,644	357,893	0,998	6,96E-03	3,86E-01
254531	LPCAT4	16,771	45,508	32,754	84,466	0,995	3,21E-02	8,46E-01
6566	SLC16A1	47,169	104,572	45,657	117,556	0,989	1,61E-02	6,04E-01
23235	SIK2	68,133	127,810	42,680	142,809	0,989	1,85E-02	6,51E-01
64764	CREB3L2	97,482	329,207	145,905	231,629	0,988	1,28E-02	5,39E-01
9587	MAD2L1BP	22,012	35,825	6,948	50,506	0,986	4,72E-02	1,00E+00
23387	SIK3	40,880	128,778	52,605	94,045	0,985	1,93E-02	6,63E-01
7422	VEGFA	759,944	1595,684	474,438	1289,635	0,985	1,16E-02	5,20E-01
59277	NTN4	29,350	76,492	26,799	62,697	0,980	2,69E-02	7,84E-01
55790	CSGALNACT1	100,627	260,461	61,538	136,713	0,977	2,54E-02	7,59E-01
50999	TMED5	42,976	122,000	43,672	85,337	0,977	2,18E-02	7,06E-01
4854	NOTCH3	128,928	297,254	132,009	293,455	0,977	8,79E-03	4,52E-01
23516	SLC39A14	263,098	602,254	105,210	325,674	0,975	2,97E-02	8,28E-01
6513	SLC2A1	214,881	674,874	335,482	552,079	0,971	9,98E-03	4,84E-01
25963	TMEM87A	60,795	129,746	55,583	141,067	0,968	1,60E-02	6,04E-01
6535	SLC6A8	30,398	42,603	24,814	101,011	0,964	3,77E-02	9,18E-01
3732	CD82	190,772	382,461	122,084	339,607	0,960	1,54E-02	5,89E-01
63893	UBE2O	45,073	111,349	44,665	98,399	0,960	2,10E-02	6,96E-01
29969	MDFIC	87,000	128,778	51,613	206,376	0,959	2,39E-02	7,39E-01
51186	TCEAL9	68,133	183,968	95,285	183,736	0,956	1,44E-02	5,73E-01
8728	ADAM19	26,205	91,984	81,389	172,416	0,951	3,80E-02	9,20E-01
23336	SYNM	24,109	45,508	38,709	113,202	0,950	4,00E-02	9,38E-01
121260	SLC15A4	36,687	90,048	33,747	74,017	0,931	3,12E-02	8,38E-01
286343	LURAP1L	35,639	68,746	19,851	67,051	0,928	4,15E-02	9,45E-01
5069	PAPPA	114,254	184,937	29,776	181,124	0,919	4,98E-02	1,00E+00
7461	CLIP2	123,687	326,302	205,458	383,146	0,917	1,62E-02	6,06E-01
256987	SERINC5	52,410	87,143	85,359	238,596	0,914	4,03E-02	9,38E-01
90	ACVR1	81,759	160,730	79,404	192,444	0,912	1,92E-02	6,63E-01
131578	LRRC15	81,759	134,587	77,419	221,180	0,911	2,38E-02	7,38E-01
7040	TGFB1	343,809	583,858	264,018	735,815	0,910	1,46E-02	5,73E-01
490	ATP2B1	47,169	119,095	34,739	70,534	0,904	4,17E-02	9,45E-01
483	ATP1B3	83,856	172,349	76,426	175,899	0,902	2,11E-02	6,96E-01
9188	DDX21	71,277	186,873	108,188	196,798	0,896	2,22E-02	7,06E-01
7035	TFPI	83,856	144,270	41,687	141,938	0,887	4,01E-02	9,38E-01
3572	IL6ST	626,823	883,048	482,379	1507,332	0,881	2,19E-02	7,06E-01
5209	PFKFB3	163,519	416,350	140,942	236,854	0,878	3,12E-02	8,38E-01
10867	TSPAN9	148,844	290,476	156,823	346,573	0,875	1,79E-02	6,40E-01
10458	BAIAP2	37,735	68,746	30,769	84,466	0,871	4,48E-02	9,83E-01
23710	GABARAPL1	87,000	190,746	78,411	156,742	0,859	3,06E-02	8,28E-01
205	AK4	28,301	66,810	38,709	79,242	0,855	4,87E-02	1,00E+00
4814	NINJ1	199,158	453,143	150,867	281,264	0,851	3,54E-02	8,99E-01
84255	SLC37A3	32,494	77,460	41,687	81,854	0,850	4,65E-02	1,00E+00
60485	SAV1	54,506	121,032	51,613	101,882	0,841	4,18E-02	9,45E-01
23541	SEC14L2	38,783	75,524	36,724	86,208	0,839	4,90E-02	1,00E+00
57403	RAB22A	34,591	97,794	50,620	81,854	0,837	4,96E-02	1,00E+00
55603	FAM46A	75,470	148,143	103,225	219,438	0,833	3,54E-02	8,99E-01

9689	BZW1	105,868	156,857	101,240	275,169	0,831	3,79E-02	9,18E-01
5467	PPARD	66,036	141,365	73,449	143,680	0,830	3,58E-02	8,99E-01
9804	TOMM20	67,085	143,302	88,337	170,674	0,819	3,74E-02	9,18E-01
54477	PLEKHA5	66,036	113,286	71,464	168,933	0,816	4,29E-02	9,57E-01
23481	PES1	58,699	127,810	67,493	128,006	0,816	4,15E-02	9,45E-01
57003	CCDC47	103,772	213,984	103,225	200,281	0,816	3,34E-02	8,66E-01
5151	PDE8A	89,097	184,937	64,516	132,360	0,814	4,99E-02	1,00E+00
1910	EDNRB	158,278	234,318	194,540	472,837	0,789	4,84E-02	1,00E+00
3091	HIF1A	231,652	375,683	276,921	621,742	0,788	3,69E-02	9,15E-01
8662	EIF3B	163,519	252,715	126,054	313,483	0,772	4,48E-02	9,83E-01
1983	EIF5	132,073	289,508	153,845	256,011	0,771	4,04E-02	9,38E-01
7291	TWIST1	115,302	224,635	111,165	208,989	0,762	4,61E-02	9,98E-01
23243	ANKRD28	93,290	192,683	133,002	238,596	0,759	4,87E-02	1,00E+00
80381	CD276	171,905	344,699	205,458	351,798	0,736	4,43E-02	9,81E-01
960	CD44	933,945	1610,208	690,814	1421,995	0,733	4,85E-02	1,00E+00
966	CD59	538,774	1139,636	723,568	1141,601	0,723	4,11E-02	9,45E-01
7162	TPBG	263,098	177,191	325,556	147,163	-0,745	4,80E-02	1,00E+00
2316	FLNA	3367,861	2115,637	4362,253	2223,989	-0,750	3,05E-02	8,28E-01
56731	SLC2A4RG	160,374	89,079	166,748	87,949	-0,766	4,83E-02	1,00E+00
57674	RNF213	448,629	243,032	329,526	166,320	-0,793	4,61E-02	9,98E-01
4641	MYO1C	488,460	295,318	571,708	270,815	-0,801	2,64E-02	7,74E-01
283078	MKX	105,868	48,413	124,069	69,663	-0,811	4,71E-02	1,00E+00
440275	EIF2AK4	114,254	68,746	145,905	64,438	-0,813	4,24E-02	9,51E-01
8573	CASK	99,579	43,571	120,098	67,921	-0,818	4,81E-02	1,00E+00
254102	EHBP1L1	278,821	148,143	282,876	146,292	-0,821	2,73E-02	7,84E-01
80332	ADAM33	132,073	68,746	135,979	68,792	-0,821	3,85E-02	9,28E-01
6938	TCF12	166,664	91,984	212,405	103,624	-0,824	3,18E-02	8,45E-01
311	ANXA11	287,206	162,667	266,003	120,169	-0,832	3,19E-02	8,46E-01
5156	PDGFRA	896,210	523,826	1184,111	559,045	-0,839	1,75E-02	6,28E-01
8476	CDC42BPA	100,627	64,873	162,778	65,309	-0,839	3,98E-02	9,38E-01
142	PARP1	123,687	70,683	141,934	61,826	-0,845	3,48E-02	8,91E-01
122953	JDP2	113,205	51,318	99,255	53,989	-0,850	3,89E-02	9,29E-01
10512	SEMA3C	337,520	187,841	245,160	94,045	-0,851	4,47E-02	9,83E-01
59	ACTA2	67,085	28,079	63,523	33,961	-0,860	4,95E-02	1,00E+00
152503	SH3D19	118,446	61,968	111,165	49,635	-0,865	3,58E-02	8,99E-01
3667	IRS1	244,230	118,127	278,906	145,421	-0,869	2,01E-02	6,74E-01
5865	RAB3B	126,832	75,524	181,636	74,888	-0,872	2,80E-02	7,95E-01
1848	DUSP6	140,459	86,175	313,645	128,876	-0,874	3,71E-02	9,15E-01
57060	PCBP4	89,097	31,952	153,845	79,242	-0,887	4,64E-02	1,00E+00
71	ACTG1	2637,267	1092,191	4142,899	2222,247	-0,888	2,40E-02	7,39E-01
23264	ZC3H7B	515,714	301,127	638,209	271,685	-0,889	1,40E-02	5,68E-01
4141	MARS	174,001	88,111	133,002	56,601	-0,892	3,33E-02	8,66E-01
115207	KCTD12	535,629	275,953	968,728	450,197	-0,895	2,22E-02	7,06E-01
64780	MICAL1	81,759	27,111	113,151	61,826	-0,900	4,15E-02	9,45E-01
23211	ZC3H4	47,169	19,365	63,523	29,607	-0,903	4,69E-02	1,00E+00
81603	TRIM8	268,339	125,873	279,899	141,938	-0,910	1,44E-02	5,73E-01
3275	PRMT2	136,266	77,460	157,815	60,955	-0,911	2,32E-02	7,23E-01
51564	HDAC7	123,687	64,873	181,636	79,242	-0,912	2,24E-02	7,06E-01
678	ZFP36L2	138,362	77,460	146,897	55,730	-0,913	2,48E-02	7,46E-01
23576	DDAH1	53,458	34,857	173,696	60,955	-0,914	4,91E-02	1,00E+00
440	ASNS	33,542	17,429	50,620	17,416	-0,925	5,00E-02	1,00E+00
400550	FENDRR	143,603	84,238	226,301	87,949	-0,928	1,89E-02	6,58E-01
54862	CC2D1A	46,121	14,524	36,724	20,028	-0,930	4,97E-02	1,00E+00
1841	DTYMK	56,603	22,270	42,680	20,028	-0,931	4,43E-02	9,81E-01
221	ALDH3B1	95,386	30,984	35,732	22,640	-0,941	5,00E-02	1,00E+00
16	AARS	210,688	91,984	146,897	72,275	-0,942	2,17E-02	7,06E-01
51115	RMDN1	48,217	23,238	57,568	21,770	-0,942	3,77E-02	9,18E-01
22836	RHOBTB3	84,904	51,318	116,128	37,444	-0,947	2,57E-02	7,65E-01
26227	PHGDH	107,964	40,667	89,329	47,893	-0,957	2,25E-02	7,06E-01

4171	MCM2	85,952	28,079	35,732	20,899	-0,960	4,46E-02	9,83E-01
1969	EPHA2	89,097	55,191	149,875	49,635	-0,962	2,15E-02	7,03E-01
64859	NABP1	50,314	36,794	151,860	45,281	-0,963	3,57E-02	8,99E-01
7402	UTRN	76,518	40,667	259,055	94,045	-0,964	3,91E-02	9,32E-01
81563	C1ORF21	37,735	26,143	86,352	23,511	-0,966	3,75E-02	9,18E-01
9400	RECQL5	29,350	13,556	36,724	11,320	-0,973	4,61E-02	9,98E-01
8989	TRPA1	101,675	43,571	61,538	23,511	-0,974	3,40E-02	8,77E-01
152573	SHISA3	0,000	0,000	20,844	0,000	-0,976	4,77E-02	NA
5921	RASA1	105,868	56,159	213,398	81,854	-0,978	2,02E-02	6,75E-01
653513	LOC653513	14,675	0,000	2,978	0,000	-0,979	4,94E-02	NA
359845	RFLNB	95,386	54,222	288,832	102,753	-0,979	3,03E-02	8,28E-01
388650	FAM69A	57,651	30,016	67,493	21,770	-0,980	2,89E-02	8,12E-01
114876	OSBPL1A	45,073	21,302	66,501	24,382	-0,981	3,06E-02	8,28E-01
11234	HPS5	37,735	15,492	28,784	8,708	-0,984	4,68E-02	1,00E+00
54858	PGPEP1	27,253	11,619	41,687	13,933	-0,991	4,18E-02	9,45E-01
29028	ATAD2	36,687	23,238	68,486	17,416	-0,995	3,38E-02	8,72E-01
23179	RGL1	79,663	18,397	54,590	36,573	-0,995	3,05E-02	8,28E-01
55619	DOCK10	58,699	24,206	95,285	39,185	-0,999	2,43E-02	7,44E-01
93627	TBCK	16,771	5,810	41,687	13,062	-1,003	4,92E-02	1,00E+00
523	ATP6V1A	71,277	43,571	136,972	42,669	-1,004	1,97E-02	6,64E-01
79924	adm2	13,627	0,000	3,970	0,000	-1,005	4,49E-02	NA
134957	STXBP5	40,880	22,270	47,642	10,449	-1,005	3,70E-02	9,15E-01
55901	THSD1	4,193	0,000	13,896	0,000	-1,008	4,46E-02	NA
84162	KIAA1109	89,097	42,603	127,046	49,635	-1,008	1,50E-02	5,86E-01
727800	RNF208	14,675	0,968	4,963	1,742	-1,009	4,79E-02	NA
64770	CCDC14	42,976	15,492	58,560	24,382	-1,009	2,92E-02	8,21E-01
6563	SLC14A1	340,664	131,683	117,121	34,831	-1,011	3,88E-02	9,29E-01
10827	FAM114A2	20,964	7,746	24,814	6,966	-1,013	4,69E-02	1,00E+00
4013	VWA5A	75,470	28,079	248,137	92,303	-1,014	3,42E-02	8,79E-01
81493	SYNC	72,326	21,302	25,806	13,933	-1,015	4,02E-02	9,38E-01
79885	HDAC11	28,301	1,937	27,791	14,803	-1,020	4,89E-02	1,00E+00
3516	RBPJ	171,905	63,905	260,048	121,039	-1,020	1,27E-02	5,39E-01
10602	CDC42EP3	59,747	42,603	126,054	30,478	-1,021	2,25E-02	7,06E-01
54532	USP53	27,253	11,619	63,523	20,899	-1,022	3,56E-02	8,99E-01
79690	GAL3ST4	25,157	7,746	28,784	10,449	-1,025	4,07E-02	9,43E-01
79890	RIN3	32,494	7,746	21,836	10,449	-1,027	4,15E-02	9,45E-01
388963	C2ORF81	12,578	1,937	12,903	3,483	-1,028	4,94E-02	1,00E+00
374393	FAM111B	25,157	2,905	11,911	6,966	-1,030	4,88E-02	1,00E+00
2139	EYA2	19,916	5,810	38,709	13,062	-1,032	4,16E-02	9,45E-01
5860	QDPR	42,976	11,619	31,762	15,674	-1,032	3,29E-02	8,60E-01
421	ARVCF	4,193	3,873	32,754	3,483	-1,033	4,83E-02	1,00E+00
64388	GREM2	319,700	150,079	186,599	26,124	-1,033	3,53E-02	8,99E-01
55103	RALGPS2	30,398	10,651	88,337	30,478	-1,033	3,59E-02	9,01E-01
6574	SLC20A1	278,821	114,254	162,778	67,051	-1,037	1,61E-02	6,04E-01
25802	LMOD1	12,578	3,873	44,665	12,191	-1,038	4,53E-02	9,91E-01
154091	SLC2A12	10,482	0,968	6,948	0,000	-1,042	3,93E-02	NA
5257	PHKB	40,880	11,619	51,613	22,640	-1,045	2,73E-02	7,85E-01
221895	JAZF1	11,530	7,746	54,590	11,320	-1,046	4,23E-02	9,51E-01
6773	STAT2	278,821	99,730	154,838	80,983	-1,046	1,29E-02	5,39E-01
83660	TLN2	58,699	20,333	158,808	59,213	-1,048	2,72E-02	7,84E-01
6935	ZEB1	124,736	57,127	248,137	94,916	-1,048	1,29E-02	5,39E-01
3985	LIMK2	38,783	11,619	32,754	13,933	-1,048	3,04E-02	8,28E-01
57484	RNF150	46,121	20,333	59,553	20,028	-1,050	2,16E-02	7,03E-01
23536	ADAT1	24,109	5,810	20,844	7,837	-1,054	3,95E-02	9,34E-01
6742	SSBP1	18,868	3,873	15,881	5,225	-1,059	4,21E-02	9,49E-01
5905	RANGAP1	286,158	126,841	326,549	137,584	-1,061	4,13E-03	2,76E-01
5063	PAK3	22,012	1,937	11,911	6,096	-1,062	4,26E-02	9,52E-01
79960	JADE1	47,169	12,587	86,352	35,702	-1,064	2,51E-02	7,52E-01
330	BIRC3	94,338	32,921	34,739	5,225	-1,066	3,76E-02	9,18E-01

85379	KIAA1671	25,157	6,778	11,911	1,742	-1,067	4,17E-02	9,45E-01
80169	CTC1	6,289	0,968	20,844	3,483	-1,067	4,01E-02	9,38E-01
390	RND3	469,593	237,222	428,781	129,747	-1,071	8,12E-03	4,27E-01
144363	ETFRF1	4,193	1,937	21,836	1,742	-1,073	3,78E-02	9,18E-01
114796	PSMG3-AS1	20,964	6,778	17,866	3,483	-1,074	3,88E-02	9,29E-01
9569	GTF2IRD1	52,410	12,587	28,784	15,674	-1,075	2,72E-02	7,84E-01
11046	SLC35D2	13,627	7,746	49,627	10,449	-1,077	3,53E-02	8,99E-01
2825	GPR1	23,060	7,746	27,791	7,837	-1,083	3,21E-02	8,46E-01
9577	BRE	16,771	5,810	23,821	5,225	-1,084	3,61E-02	9,01E-01
115557	ARHGEF25	89,097	30,016	75,434	34,831	-1,084	1,19E-02	5,27E-01
862	RUNX1T1	8,386	0,000	9,925	0,000	-1,085	3,28E-02	NA
5783	PTPN13	159,326	81,333	262,033	90,562	-1,085	6,04E-03	3,46E-01
91624	NEXN	23,060	11,619	88,337	23,511	-1,091	2,83E-02	7,98E-01
219285	SAMD9L	78,615	31,952	54,590	15,674	-1,094	1,95E-02	6,64E-01
10669	CGREF1	14,675	1,937	6,948	0,000	-1,094	3,36E-02	NA
84939	MUM1	81,759	22,270	88,337	42,669	-1,105	1,19E-02	5,27E-01
60686	C14ORF93	10,482	2,905	15,881	1,742	-1,112	3,35E-02	8,68E-01
388115	C15orf52	56,603	22,270	35,732	4,354	-1,117	2,80E-02	7,95E-01
57523	NYNRIN	37,735	15,492	52,605	15,674	-1,117	1,75E-02	6,28E-01
6339	SCNN1D	15,723	0,000	15,881	5,225	-1,124	3,20E-02	8,46E-01
55793	FAM63A	20,964	5,810	18,858	4,354	-1,125	3,00E-02	8,28E-01
84795	PYROXD2	29,350	3,873	34,739	14,803	-1,128	2,61E-02	7,72E-01
55614	KIF16B	16,771	6,778	21,836	1,742	-1,129	3,08E-02	8,30E-01
29128	UHRF1	59,747	12,587	48,635	25,253	-1,131	1,58E-02	6,00E-01
2027	ENO3	12,578	0,000	6,948	0,000	-1,132	2,63E-02	NA
23051	ZHX3	98,531	44,540	114,143	36,573	-1,133	6,68E-03	3,73E-01
55388	MCM10	19,916	4,841	11,911	0,000	-1,134	3,03E-02	8,28E-01
4061	LY6E	32,494	11,619	23,821	0,871	-1,135	2,99E-02	8,28E-01
7791	ZYX	236,893	98,762	353,347	135,843	-1,139	3,10E-03	2,38E-01
596	BCL2	14,675	1,937	13,896	3,483	-1,139	2,97E-02	8,28E-01
5021	OXTR	19,916	1,937	5,955	1,742	-1,140	2,81E-02	7,95E-01
387758	FIBIN	33,542	9,683	39,702	13,933	-1,140	1,87E-02	6,56E-01
79443	FYCO1	149,892	54,222	195,532	80,983	-1,141	4,28E-03	2,82E-01
10245	TIMM17B	20,964	6,778	30,769	7,837	-1,141	2,44E-02	7,44E-01
291	SLC25A4	27,253	4,841	21,836	8,708	-1,143	2,53E-02	7,57E-01
84171	LOXL4	63,940	24,206	43,672	10,449	-1,150	1,71E-02	6,26E-01
9223	MAGI1	47,169	17,429	68,486	22,640	-1,156	1,13E-02	5,17E-01
5137	PDE1C	32,494	5,810	7,940	1,742	-1,157	2,73E-02	7,84E-01
5828	PEX2	42,976	15,492	66,501	21,770	-1,157	1,23E-02	5,34E-01
54899	PXK	41,928	14,524	65,508	21,770	-1,158	1,27E-02	5,39E-01
23022	PALLD	176,097	95,857	328,534	94,916	-1,162	3,71E-03	2,64E-01
3306	HSPA2	48,217	23,238	78,411	20,028	-1,167	1,02E-02	4,90E-01
23371	TNS2	107,964	28,079	70,471	36,573	-1,168	7,94E-03	4,23E-01
5939	RBMS2	77,567	24,206	74,441	30,478	-1,174	7,04E-03	3,89E-01
2115	ETV1	37,735	13,556	128,039	36,573	-1,179	1,57E-02	6,00E-01
81848	SPRY4	47,169	23,238	70,471	15,674	-1,180	1,06E-02	4,99E-01
9260	PDLIM7	227,459	98,762	423,818	140,197	-1,207	2,48E-03	2,07E-01
2992	GYG1	38,783	7,746	29,776	12,191	-1,208	1,45E-02	5,73E-01
24139	EML2	48,217	10,651	43,672	18,287	-1,210	1,08E-02	5,04E-01
10507	SEMA4D	17,819	1,937	8,933	1,742	-1,217	1,97E-02	6,64E-01
55504	TNFRSF19	202,302	120,064	303,720	49,635	-1,218	5,30E-03	3,19E-01
27147	DENND2A	71,277	15,492	34,739	17,416	-1,218	1,11E-02	5,09E-01
10611	PDLIM5	102,723	56,159	253,100	65,309	-1,219	4,68E-03	2,97E-01
84189	SLITRK6	19,916	2,905	9,925	1,742	-1,221	1,97E-02	6,64E-01
57724	EPG5	73,374	24,206	64,516	21,770	-1,233	5,62E-03	3,28E-01
60	ACTB	2728,460	1067,985	3786,574	1397,612	-1,241	4,68E-04	6,40E-02
171024	SYNPO2	29,350	24,206	111,165	13,933	-1,241	1,16E-02	5,20E-01
1958	EGR1	127,880	45,508	474,438	135,843	-1,241	8,88E-03	4,53E-01
1902	LPAR1	546,111	200,429	530,021	205,506	-1,241	6,04E-04	7,65E-02

64750	SMURF2	165,615	36,794	138,957	70,534	-1,242	3,18E-03	2,42E-01
7846	TUBA1A	312,363	101,667	856,570	276,039	-1,247	5,66E-03	3,28E-01
64221	ROBO3	28,301	9,683	52,605	13,062	-1,252	1,02E-02	4,90E-01
154796	AMOT	32,494	20,333	155,830	31,348	-1,254	1,04E-02	4,93E-01
112574	SNX18	150,941	45,508	199,502	77,500	-1,258	1,98E-03	1,75E-01
147495	APCDD1	78,615	38,730	198,510	49,635	-1,271	3,99E-03	2,75E-01
8507	ENC1	62,892	25,175	75,434	18,287	-1,279	4,36E-03	2,84E-01
23677	SH3BP4	137,314	53,254	157,815	48,764	-1,279	1,42E-03	1,44E-01
4329	ALDH6A1	13,627	3,873	25,806	3,483	-1,280	1,41E-02	5,70E-01
54328	GPR173	12,578	3,873	24,814	2,612	-1,282	1,42E-02	5,72E-01
6869	TACR1	30,398	5,810	12,903	1,742	-1,289	1,38E-02	5,64E-01
57761	TRIB3	56,603	9,683	25,806	12,191	-1,293	9,08E-03	4,57E-01
92154	MTSS1L	235,845	93,921	247,145	73,146	-1,298	8,49E-04	9,74E-02
5393	EXOSC9	15,723	2,905	26,799	5,225	-1,305	1,21E-02	5,31E-01
9742	IFT140	63,940	16,460	61,538	21,770	-1,315	3,61E-03	2,62E-01
2491	CENPI	8,386	1,937	19,851	0,000	-1,316	1,15E-02	5,20E-01
4857	NOVA1	20,964	1,937	18,858	5,225	-1,318	1,16E-02	5,20E-01
116832	RPL39L	19,916	3,873	27,791	6,096	-1,322	1,02E-02	4,90E-01
9241	NOG	12,578	0,000	13,896	1,742	-1,328	1,08E-02	5,04E-01
2250	FGF5	32,494	14,524	134,987	27,865	-1,332	6,57E-03	3,69E-01
323	APBB2	31,446	11,619	39,702	5,225	-1,343	7,17E-03	3,92E-01
26230	TIAM2	25,157	3,873	20,844	5,225	-1,353	8,72E-03	4,50E-01
4008	LMO7	109,013	82,302	459,550	74,017	-1,354	3,35E-03	2,51E-01
10641	NPRL2	20,964	0,000	12,903	3,483	-1,359	9,49E-03	4,73E-01
6218	RPS17	32,494	3,873	77,419	20,899	-1,360	7,09E-03	3,90E-01
64900	LPIN3	27,253	9,683	43,672	6,966	-1,369	5,81E-03	3,36E-01
158056	MAMDC4	33,542	7,746	36,724	8,708	-1,405	4,35E-03	2,84E-01
9020	MAP3K14	22,012	5,810	25,806	2,612	-1,411	6,41E-03	3,62E-01
1265	CNN2	272,532	103,603	671,956	174,157	-1,414	8,14E-04	9,61E-02
9659	PDE4DIP	101,675	27,111	78,411	24,382	-1,427	1,10E-03	1,19E-01
5924	RASGRF2	56,603	19,365	76,426	16,545	-1,429	1,58E-03	1,52E-01
9760	TOX	6,289	0,000	23,821	0,000	-1,435	5,66E-03	3,28E-01
22874	PLEKHA6	42,976	10,651	30,769	5,225	-1,435	4,11E-03	2,76E-01
25959	KANK2	361,628	121,032	517,118	155,000	-1,442	1,14E-04	2,10E-02
9079	LDB2	33,542	12,587	59,553	8,708	-1,458	2,63E-03	2,13E-01
347902	AMIGO2	11,530	3,873	72,456	8,708	-1,493	4,05E-03	2,75E-01
84962	AJUBA	42,976	4,841	63,523	17,416	-1,531	1,80E-03	1,66E-01
57608	KIAA1462	281,965	106,508	320,593	54,860	-1,540	1,98E-04	3,33E-02
79633	FAT4	129,977	25,175	93,300	33,090	-1,553	3,15E-04	4,76E-02
3790	KCNS3	12,578	1,937	31,762	2,612	-1,603	2,18E-03	1,88E-01
23566	LPAR3	16,771	0,000	17,866	1,742	-1,609	2,13E-03	1,86E-01
2353	FOS	18,868	13,556	204,465	19,157	-1,621	1,56E-03	1,52E-01
54510	PCDH18	805,016	140,397	871,458	304,775	-1,623	4,21E-05	9,61E-03
22861	NLRP1	27,253	4,841	25,806	1,742	-1,663	1,31E-03	1,35E-01
27123	DKK2	27,253	3,873	39,702	6,096	-1,700	7,60E-04	9,29E-02
10129	FRY	6,289	0,000	38,709	0,000	-1,718	1,00E-03	1,13E-01
79094	CHAC1	27,253	0,968	13,896	1,742	-1,754	8,14E-04	9,61E-02
113146	AHNAK2	32,494	1,937	49,627	8,708	-1,808	3,56E-04	5,18E-02
10290	SPEG	116,350	16,460	150,867	37,444	-1,814	3,30E-05	8,08E-03
55784	MCTP2	73,374	20,333	146,897	21,770	-1,825	3,92E-05	9,15E-03
79640	C22ORF46	35,639	5,810	36,724	2,612	-1,888	1,90E-04	3,30E-02
4739	NEDD9	10,482	0,000	46,650	0,871	-2,032	1,06E-04	1,97E-02
89795	NAV3	105,868	8,714	42,680	8,708	-2,110	1,45E-05	4,03E-03
64283	ARHGEF28	34,591	5,810	64,516	1,742	-2,238	8,84E-06	2,68E-03
652	BMP4	63,940	6,778	69,478	5,225	-2,421	3,30E-07	1,54E-04
1490	CTGF	170,856	52,286	1006,445	16,545	-2,599	1,55E-07	8,39E-05
3491	CYR61	684,473	16,460	1222,820	8,708	-4,862	3,86E-31	3,97E-27
